# Prevalence, burden of disease, and lost in health state utilities attributable to chronic musculoskeletal disorders and pain in Chile

**DOI:** 10.1186/s12889-021-10953-z

**Published:** 2021-05-17

**Authors:** Pedro Zitko, Norberto Bilbeny, Carlos Balmaceda, Tomas Abbott, Cesar Carcamo, Manuel Espinoza

**Affiliations:** 1 Department of Heath Service & Population Research, IoPPN, Kings College London, London, UK; 2 Departamento de Salud Pública, Pontificia Universidad Católica de Chile, Diagonal Paraguay, 362, Piso 2, Santiago, Chile; 3 Asociación Chilena para el Estudio del Dolor, Santiago, Chile; 4 Unidad de Evaluación de Tecnologías Sanitarias, Centro de Investigación Clínica, Pontificia Universidad Católica de Chile, Santiago, Chile

**Keywords:** Burden of disease, Pain, Musculoskeletal diseases, Prevalence, Outcome measures

## Abstract

**Background:**

Musculoskeletal disorders are a leading cause of disability adjusted life years (DALY) in the world. We aim to describe the prevalence and to compare the DALYs and loss of health state utilities (LHSU) attributable to common musculoskeletal disorders in Chile.

**Methods:**

We used data from the Chilean National Health Survey carried out in 2016–2017. Six musculoskeletal disorders were detected through the COPCOPRD questionnaire: chronic musculoskeletal pain, chronic low back pain, chronic shoulder pain, osteoarthritis of hip and knee, and fibromyalgia. We calculated the DALY for each disorder for 18 sex and age strata, and LHSU following an individual and population level approaches. We also calculated the fraction of LHSU attributable to pain.

**Results:**

Chronic musculoskeletal pain disorder affects a fifth of the adult population, with a significant difference between sexes. Among specific musculoskeletal disorders highlights chronic low back pain with the highest prevalence. Musculoskeletal disorders are a significant cause of LHSU at the individual level, especially in the case of fibromyalgia. Chronic musculoskeletal pain caused 503,919 [283,940 - 815,132] DALYs in 2017, and roughly two hundred thousand LSHU at population level, which represents 9.7% [8.8–10.6] of the total LSHU occurred in that year. Discrepancy in the burden of musculoskeletal disorders was observed according to DALY or LSHU estimation. The pain and discomfort domain of LHSU accounted for around half of total LHSU in people with musculoskeletal disorders.

**Conclusion:**

Chronic musculoskeletal pain is a major source of burden and LHSU. Fibromyalgia should deserve more attention in future studies. Using the attributable fraction offers a straightforward and flexible way to explore the burden of musculoskeletal disorders.

**Supplementary Information:**

The online version contains supplementary material available at 10.1186/s12889-021-10953-z.

## Background

Musculoskeletal disorders and the pain associated with those conditions, are one of the leading causes of burden of disease in the world. Estimates from the last Burden of Disease Study points out that musculoskeletal disorders accounted globally, in 2017, for 139 million disability adjusted life years (DALY), highlighting low back pain with 64.9 million DALY and osteoarthritis with 9.6 millions DALY [1]. In particular, more than 95% of this burden is due to disability [2].

DALYs is a very well-known and accepted metric that has been used for a long time ago. It has the advantage to combine deaths and disability outcomes in a single indicator. The DALYs are expressed in units of time, which make them easily understood by decision-makers. Additionally, it has been used for multiple purposes: setting health service priorities, inequities analysis, assessing health consequences from healthcare interventions, and identifying knowledge gaps [3] . Furthermore, several countries have conducted their own studies of the burden of diseases using DALYs, which promote the comparability of results. Nevertheless, DALYs are not the only possible approach, there are other methodologies capable to estimate the impact of diseases in populations, which are less studied, including in the case of musculoskeletal disorders [4]. One of these approaches is based on the epidemiological concept of population attributable fraction [5, 6]. When the population attributable fraction is calculated on an outcome such as disability, it can inform about the fraction of disability in a population that is attributable to a certain disease. This method offers a very straightforward and flexible approach to explore the burden of disease and is suitable to be applied to data collected from national health surveys, especially for chronic non-communicable conditions [6]. Moreover, this approach overcomes some of the common critics to the calculus of DALY, such as the use of foreign disability weights, very sophisticated methodological procedures which preclude to disseminate its use across governments, and the use of a complex and limited techniques to adjust the burden of a disease by comorbidities [7].

The procedure of attributable fraction also allows to be applied to different outcomes than disability. Another approach used to characterize the health status of population, which are suitable for decision making analysis, is through health state utilities (HSU) [8]. The HSU are used in the calculus of the quality adjusted life years (QALY), a well-accepted metric commonly used in cost-effective and cost-utility analysis [9].

Since DALYs are based on disabilities, and HSU are based on social preferences about health states, usually both approaches are presented as opposite methodological strategies to orient decision makers [10, 11].

In this study we estimate the prevalence and burden of common chronic musculoskeletal disorders and the pain associated to them, in Chile, by using both methodological approaches, DALYs and Lost of HSU (LHSU) base on attributional fractions. This comparison allows to generate insight about both procedures, knowing the extent they coincide prioritizing health conditions, and how their results can complement each other. We also take advantage of the flexibility of attribution methods to describe the LHSU by domain of functioning, with special focus on pain.

## Methods

### Sample

We used data from the third Chilean National Health Survey carried out between August 2016 and March 2017 (ChNHS 2016–2017). It corresponds to a nationally representative sample of people older than 15 years, structured as a multistage complex design. Interviews and collection of laboratory and anthropometric measures were accomplished by trained individuals in at least two visits to the household of participants. Several quality control check points were implemented. The overall response rate was 67.0%, which corresponds to 6233 respondents. The survey was commanded by the Chilean Ministry of Health and performed by the Department of Public Health of the Pontificia Universidad Católica de Chile with ethical approval from the Ethic Committee of the same university.

### Musculoskeletal disorders and pain

We included six major musculoskeletal disorders associated with chronic pain, all capable to be identified through the ChNHS 2016–2017, namely: chronic low back pain, chronic shoulder pain, hip and knee osteoarthritis, fibromyalgia and chronic musculoskeletal pain. The latest is a broad diagnosis which encompasses multiple musculoskeletal disorders and was recently added to the 11th version of the International Classification of Diseases [12]. The main questionnaire used in this survey to identify musculoskeletal disorders was the Community Oriented Programme for the Control of Rheumatic Disease Core Questionnaire (COPCORD-CQ) [13]. This instrument collects information about the presence of ‘pain, stiffness, sensitivity or bone, muscle or joints swelling’ in 22 body regions, a cardinal symptom of musculoskeletal conditions. All musculoskeletal disorders considered only cases with pain during the last 7 days lasting at least 3 months in the body region of the disorder. Since the respondents could point out different pain locations, for chronic low back pain, chronic shoulder pain, and hip and knee osteoarthritis, we choose only cases with a declared preferential location of pain in the body region of the disorder. For chronic musculoskeletal pain disorder, we selected cases with pain in any of the 22 locations explored by the COPCORD-CQ, restricting the cases to those with intensity of pain ≥3/10 using a visual analogue scale. For fibromyalgia, hip and knee osteoarthritis, cases associated to a traumatic cause of pain were excluded. For fibromyalgia we attempted to meet the American College of Rheumatology 2010 criteria [14]. However, since these criteria include symptoms that were not explored by COPCORD-CQ, we were forced to use other questionnaires available in the ChNHS 2016–2017 to fulfil them. Cognitive symptoms, unrefreshed sleep and somatic symptoms were extracted from items of a disability questionnaire, which asks the following questions: ‘Due to your health, how difficult was to remember things or concentrate?’, ‘Due to your health, how difficult is it to sleep?’ and ‘Due to your health, how much difficulty did you have in feeling any physical pain, such as back pain, stomach pain, or headache?’, respectively [15]. Fatigue symptoms were extracted from an item available in the CIDI-SF questionnaire (see below), which formulates the following questions: ‘During those same two weeks [of depressive symptoms], did you become more tired or with less energy than usual?’. Fibromyalgia was defined as ≥7 pain locations and a score ≥ 5 for other symptoms, or between 3 and 6 pain locations and a score ≥ 9 for other symptoms, restricting cases to those who had pain intensity ≥3/10.

### Other variables used in the analysis

For description and adjustment purposes, we included in the analysis other variables extracted from the ChNHS 2016–2017. They include age; sex; marital status (married/cohabiting, annulled/separated/divorced, widowed, single); education (more than 12 years, between 9 and 12 years, less than 9 years of formal schooling); working status (working for salary, looking for work, working without salary, and not working and not looking for); and three prevalent comorbidities, hypertension, diabetes and depression, all of them frequently associated to musculoskeletal disorders [16]. Hypertension was defined as blood pressure ≥ 140 mmHg systolic and/or ≥ 90 mmHg diastolic after five minutes of rest, or normal blood pressure but self-report of diagnosis and treatment (i.e., lifestyle modifications or under drug treatment). Similarly, diabetes was defined as fasten glycemia ≥125 mg/dl or normal glycemia but self-report of diagnosis and treatment (i.e., oral hypoglycaemic agents or insulin). To detect cases with a depressive episode during the last 12 months, the survey used the Composite International Diagnosis Interview short-form (CIDI-SF) questionnaire [17, 18] applying the DSM-IV criteria [19].

### Burden of Disease

We calculated the number of Disability Adjusted Life Years (DALY) [3, 20] due to the six selected musculoskeletal disorder for the year 2017, in Chile. This metric results from the sum of years of life lost by premature death (YLL) and years lived with disability (YLD) due to a certain disease. For the set of selected musculoskeletal disorders, we assumed zero YLL, and any record of them was attributed to error of misclassification. The number of YLD were calculated through the multiplication of the number of prevalent cases with a particular sequel of the disease and a disability weight for that specific sequel (i.e., YLD_i_ = *P*_*i*_ *x D*_*i*,_ where *i* is the sequel), and then adding up the marginal results of the different sequels of the disease (i.e., ${\Sigma}_i^n$ YLD_*i*_). Disability weights were extracted from the Global Burden of Disease study [21], its values can range between 0 (absence of disability) and 1 (death), and are assumed the same across countries. The description of sequels by each musculoskeletal disorder and the disability weights are available in the supplementary material (Table S1). Smoothed prevalence of each sequel according to single ages for each sex were obtained using a backward selection method on a regression model that initially included a quadratic and cubic functional from for age and an interaction term between age and sex. Predicted values for single ages were calculated and then multiplied by the intercensal population expected for Chile in the year 2017 [22], through a Monte Carlo simulation (10,000 replications), assuming beta distribution for the prevalence [23]. The number of cases for each sequel and each single age were collapsed in 18 strata of age and sex, assuming gamma distribution. Then, the prevalence for each stratum was recalculated dividing them by the intercensal population. The adjustment by sequels was carried out using the COMO procedure described elsewhere [24]. Briefly, for each stratum we simulated a population of 5000 individuals with the probability of presenting each sequel equal to their prevalence, assuming independency between sequels. A total disability weight was calculated for each simulant and used to adjust the disability associated to each sequel. The procedure is repeated 1000 times in order to propagate the second order uncertainty around parameters, assuming beta and binomial distributions as appropriate. The result of the COMO procedure is a YLD rate, which is multiplied by the intercensal population of the strata, to obtain the YLD due to the sequel. The YLD from different sequels are added to obtain the YLD due to a musculoskeletal disorder, assuming a gamma distribution. People younger than 15 years was assumed with 0 DALYs due to musculoskeletal disorders.

### Loss in health state utilities

The health state utilities (HSU) were calculated using the EQ. 5D questionnaire, which inquiries about the statements ‘that best describe your own health state today’ through 5 items with three Likert alternatives. It explores the following domains of functioning (one item for domain): mobility, self-care, usual activities, pain/discomfort, and anxiety/depression [25]. For instance, for the pain and discomfort domain the three Likert alternatives rang from ‘I have no problem in walking around’ to ‘I am confined to bed’ .The combination of answers can describe 243 different health sates, which are transferred to a continuous scale anchored between values equal to 0 (equivalent to death) and 1 (perfect health) using social preferences previously estimated for Chilean population through a time trade-off protocol [26]. Because we are interested in exploring the loss of HSU (also referred as disutilities) we transformed the HSU according to: LHSU = (1 – HSU), where 0 means perfect health, and 1 means death. On some occasions, duly indicated, for better interpretation, we preferred to use a scale between 0 and 100 (e.g., reporting coefficients from regression models). The LHSU attributable to each musculoskeletal disorder, at individual level, was estimated using an ordinary least square regression model where the dependent variable was the LHSU and the independent variable was the disorder, adjusted or not by sociodemographic variables, other musculoskeletal disorders, and comorbidities (i.e. hypertension, diabetes and depression). Using the same regression model, we calculated the expected value of LHSU separately by people with the musculoskeletal disorder (LHSU_1_) and without (LHSU_0_). Also, we predicted values of LHSU for people with the musculoskeletal disorder assuming a counterfactual scenario where they do not have the condition, i.e., imputing zero in the dummy variable that marks the presence of the musculoskeletal disorder (LHSU_1_′). The total LHSU attributable to each musculoskeletal disorder, at population level, was calculated as the sum across *n* individuals (*i*) of the subtraction between LHSU_1*i*_ and LHSU_1 *i*_’ (i.e., ${\Sigma}_i^n$ [LHSU_1*i*_ – LHSU_1_’_*i*_]), where LHSU_1*i*_ and LHSU_1 *i*_’ are the individual predicted values of LHSU in people with the musculoskeletal disorder, and under the counterfactual scenario assuming the absence of such disorder (1’) . Finally, the fraction of the total LHSU in the population produced during 2017, attributable to each musculoskeletal disorder was calculated as: (${\Sigma}_i^n$ [LHSU_1*i*_ – LHSU_1i_’])/ (${\Sigma}_i^n$ [LHSU_1i_] + ${\Sigma}_i^n$ [LHSU_0i_]), which is equivalent to the epidemiological concept of population attributable fraction. Similar procedure but for dichotomous outcomes has been described previously [6]. The extended methodology has been recently submitted by Zitko P. et al.

### Pain domain of loss of health utilities

Since the EQ. 5D questionnaire explores the health state using different domains of functioning, and one of them is ‘pain and discomfort’, it was possible to calculate the counterfactual scenario where no one reports pain and discomfort. This was accomplished imputing the lowest value in the variable of the pain and discomfort item of the EQ. 5D, and then recalculating the LHSU. In that way it is possible to calculate the fraction of the LHSU that are attributable to the domain of pain and discomfort: (observed LHSU – LHSU assuming no pain of discomfort) / observed LHSU. The calculus can be performed in population with different musculoskeletal disorders, and for all domains of the EQ. 5D.

All statistical analysis was performed using the software R 3.5.0. All parameters were calculated considering the sample weights coming from the survey design. The main functions used to calculate the burden are available in the supplementary material.

## Results

From 6233 responders, 18.5% was eliminated from the analysis because missing values in some of the variables, principally due to the absence of glycemia and blood pressure measurement (see the supplementary material for an analysis of missing data). A description of the remaining sample (*n* = 5077) is presented in Table 1. The average of LHSU in the general population was 22.4 from a scale anchored in 0 (equivalent to perfect health) and 1 (equivalent to death). Characteristics of the population for each selected musculoskeletal disorder are shown in supplementary material. The prevalence of the musculoskeletal disorders for the whole population and also stratified by sex is presented in Table 2. Chronic musculoskeletal pain disorder affected roughly a fifth of the adult population, with a clear difference by sex. Chronic low back pain was the most frequent specific musculoskeletal disorder, both in men and women. Fibromyalgia was the second most frequent specific disorder, especially in women, followed by osteoarthritis of knee. Prevalence by sequels, and strata of age and sex is available in the supplementary material.

**Table 1:** Description of the sample. Chilean National Health Survey 2016–2017 (*n* = 5077)

	n	mean	CI
Loss of Health state Utilities (mean)	–	22.4	[21.3–23.5]
Age (mean)	–	43.3	[42.5–44.2]
	**n**	**%**	**CI**
Sex (females)	3211	51.8	[49.3–54.3]
Marita status			
*married/ cohabiting*	2425	50.4	[47.9–52.9]
*annulled/separated/divorced*	558	8.3	[7.0–9.7]
*widower*	528	5.1	[4.3–5.9]
*Single*	1566	36.2	[33.7–38.7]
Education			
*> 12 years*	1071	26.6	[24.2–28.9]
*9–12 years*	2223	48.4	[45.9–50.9]
*< 9 years*	1783	25.0	[23.1–27]
Working status			
*working for salary*	2368	53.1	[50.6–55.5]
*looking for work*	140	2.9	[2.1–3.7]
*working without salary*	1005	17.7	[15.9–19.6]
*Not working, and not looking for*	1564	26.3	[24.1–28.4]
Comorbidity			
*Hypertension*	1894	28.3	[26.1–30.4]
*Diabetes*	787	12.0	[10.5–13.5]
*Depressive episode*	722	16.8	[14.8–18.8]

*CI* confidence intervals 95%

Note: Means and percentages are estimates from the population, which consider sample weights

**Table 2:** Prevalence of musculoskeletal disorder in general population and stratified by sex. Chilean National Health Survey 2016–2017 (n = 5077)

	Both sexes		Men		Women	
	%	CI	%	CI	%	CI
Chronic low back pain	6.9	[5.4–8.3]	6.8	[4.5–9.1]	7.0	[5.1–8.8]
Chronic shoulder pain	2.8	[2.0–3.5]	1.9	[1.0–2.7]	3.6	[2.5–4.8]
Osteoarthritis of Hip	2.1	[1.6–2.6]	0.4	[0.1–0.7]	3.6	[2.7–4.5]
Osteoarthritis of Knee	3.5	[2.6–4.3]	1.5	[0.6–2.5]	5.2	[3.9–6.5]
Fibromyalgia	3.9	[3.0–4.8]	1.4	[0.4–2.4]	6.3	[4.9–7.7]
Chronic Musculoskeletal pain	21.8	[19.8–23.8]	15.2	[12.4–18.0]	27.9	[25.1–30.8]

*CI* confidence intervals 95%

The LHSU attributable to musculoskeletal disorders at individual level is shown in Table 3. After the adjustment by sociodemographic variables and other comorbidities the LHSU attributable dropped importantly in all disorders. In average, fibromyalgia was associated to a LHSU equal to 23.2 [16.7 to 29.7], in a scale anchored between 0 (i.e., perfect health) and 100 (i.e. death), far above the next disorder in decreasing order. Osteoarthritis of knee and chronic musculoskeletal pain disorder presented similar values of LHSU, followed by osteoarthritis of hip and low back pain. Chronic shoulder pain after the adjustment by covariables showed close to zero LHSU.

**Table 3:** Unadjusted and adjusted Loss of Heath State Utilities^a^ attributable to musculoskeletal disorder. Chilean National Health Survey 2016–2017 (*n* = 5077)

	Unadjusted^¶^		Adjusted^b^	
	LHSU	CI	LHSU	CI
Chronic low back pain	8.6	[4.0 to 13.2]	5.5	[1.9 to 9.1]
Chronic shoulder pain	9.7	[2.9 to 16.5]	0.5	[−6.2 to 7.2]
Osteoarthritis of Hip	27.9	[19.6 to 36.2]	7.4	[−0.4 to 15.1]
Osteoarthritis of Knee	27.0	[20.0 to 34.1]	10.1	[2.5 to 17.7]
Fibromyalgia	37.3	[30.8 to 43.7]	23.2	[16.7 to 29.7]
Chronic Musculoskeletal pain	16.0	[13.3 to 18.8]	9.9	[7.4 to 12.5]

*CI* confidence intervals 95% / *LHSU* Loss of Health State Utilities

^a^ Loss of HSU (LHSU) are anchored in values 0 and 100, equivalent to perfect health and death, respectively

^b^ Adjusted trough a multivariate regression model including age, sex, marital status, education, working status, hypertension, diabetes, depression, and musculoskeletal disorders. In the case of chronic musculoskeletal pain, the condition was not adjusted by other musculoskeletal disorders. Other musculoskeletal disorders are adjusted among themselves without including chronic musculoskeletal pain. Complete results from regression models are available in supplement material

The number of disability adjusted life years (DALY) due to musculoskeletal disorders and the total LHSU for each condition are presented in Table 4. Additionally, the table also shows the fraction of total LHSU that are attributable to each musculoskeletal disorder. Chronic musculoskeletal pain disorder accounted for approximately half million DALYs and roughly two hundred thousand of LHSU for 2017. This amount of LHSU is equivalent to approximately a tenth of total LHSU produced in the year of the study. Fibromyalgia occupied the second place in magnitude, either using DALYs or LHSU, followed by chronic low back pain. Chronic shoulder pain generated almost fifty thousand DALYs, overcoming the burden from osteoarthritis of hip and knee, while in terms of LHSU, chronic shoulder pain showed the lowest magnitude compared with the other disorders. Rate of DALYs and LHSU, and DALYs stratified by age groups and sex is available in supplementary material.

**Table 4:** Disability adjusted life years, loss of health state utilities^a^, and the fraction of health state utilities lost attributable to musculoskeletal disorder, using data from the Chilean National Health Survey 2016–2017 (*n* = 5077)

	DALY	CI	LHSU	CI	Fraction of HSU lost (%)	CI
Chronic low back pain	142,798	[100,728–194,089]	45,089	[36,718–53,414]	1.7	[1.4–2.0]
Chronic shoulder pain	41,570	[22,587–67,371]	1680	[1312–2048]	0.1	[0.0–0.1]
Osteoarthritis of Hip	10,001	[6361–14,942]	18,324	[14,857–21,771]	0.7	[0.5–0.8]
Osteoarthritis of Knee	16,315	[10,924–23,634]	41,698	[33,201–50,203]	1.5	[1.2–1.9]
Fibromyalgia	239,265	[138,883–388,334]	109,426	[89,076–129,397]	4.1	[3.3–4.8]
Chronic Musculoskeletal pain	503,919	[283,940–815,132]	259,712	[237,736–281,717]	9.7	[8.8–10.6]

*CI* confidence intervals 95% / *DALY* disability adjusted life years / *LHSU* Loss of Health State Utilities

^a^ Loss of HSU (LHSU) are anchored in values 0 and 1, equivalent to perfect health and death, respectively

**Fig. 1:**
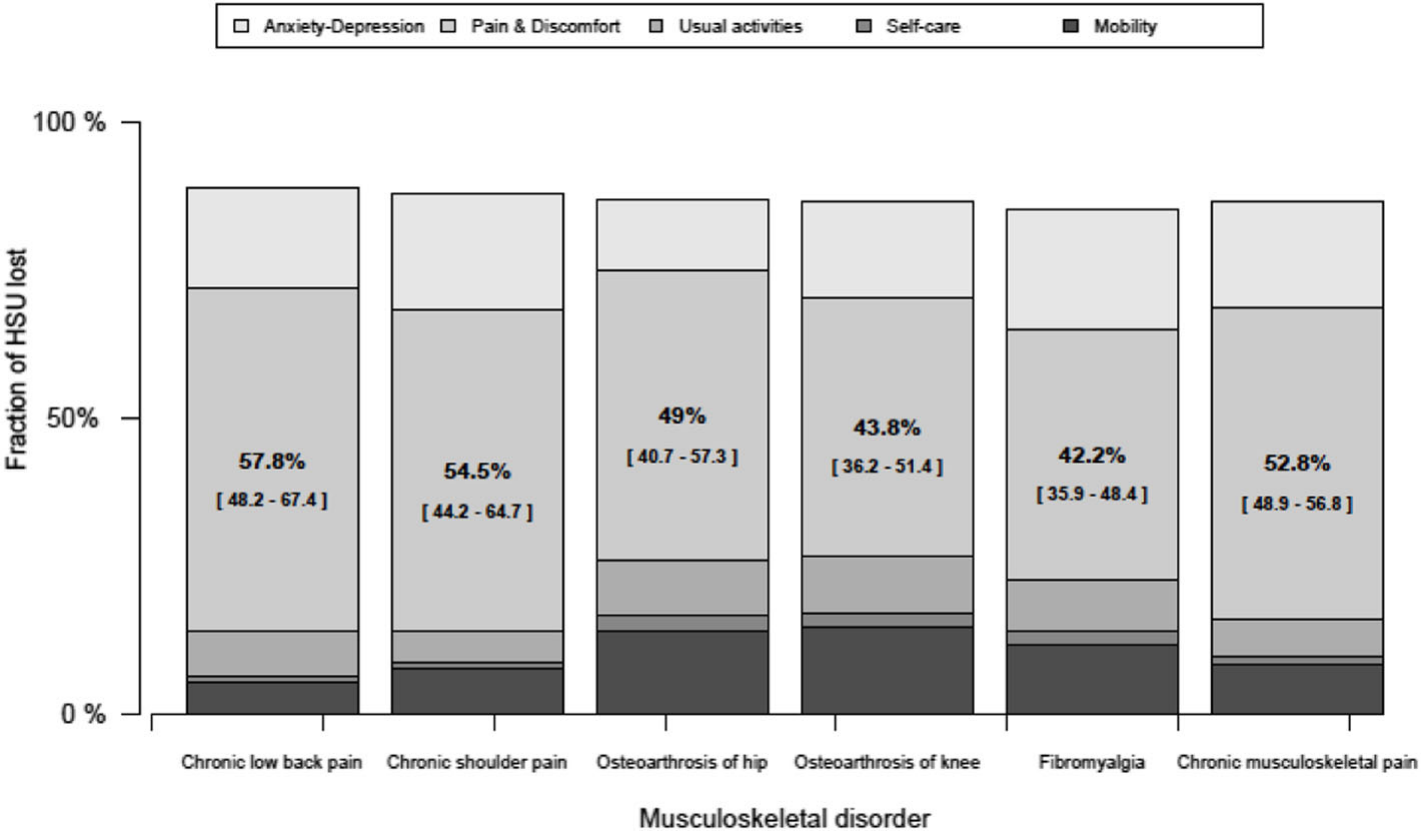
Fraction of loss of health state utilities† for musculoskeletal disorders attributable to different domains of the EQ-5D, using data from the Chilean National Health Survey 2016–2017 (*n* = 5077). Note: *HSU: Health State Utilities*. *† Loss of HSU (LHSU) are anchored in values 0 and 1, equivalent to perfect health and death, respectively. Percentages correspond to pain & discomfort domain of EQ-5D*

Finally, in Fig. 1 is shown the fraction of LHSU attributable to each musculoskeletal disorder disaggregated by the five domains of the EQ. 5D. Pain and discomfort domain accounted the highest proportion in all disorders, reaching 57.8% [48.2–67.4] for chronic low back pain. Other domains, such as mobility also was important in osteoarthritis of hip and knee, while anxiety/depression was higher in fibromyalgia. More details are available in the supplementary material.

## Discussion

Chronic musculoskeletal pain disorder affects a fifth of the adult Chilean population, with a significant difference between sexes. Among other musculoskeletal disorders highlights chronic low back pain with the highest prevalence. On the other hand, musculoskeletal disorders are a significant cause of LHSU at the individual level, especially in the case of fibromyalgia. Chronic musculoskeletal pain disorder caused half a million DALYs in 2017, and a high amount of LSHU at population level, which represents a tenth of the total LSHU occurred in that year. Discrepancy in the relevance between musculoskeletal disorders was observed between DALY and LSHU estimations. The pain and discomfort domain of LHSU accounted for around half of total LHSU in people with musculoskeletal disorders.

Observed prevalence for chronic musculoskeletal pain, chronic low back pain and osteoarthritis of hip and knee are in the range of those reported by systematic reviews for these disorders [27–32]. The prevalence of chronic low back pain has been informed between 4.2 and 10.1% [27]. The prevalence of osteoarthritis of knee and hip is very variable according the method of detection. For osteoarthritis of knee, studies using a symptomatic approach has evidenced prevalence between 6.6 and 24.2%, however these studies are usually restricted to older population [28]. Another systematic review reported a global prevalence equal to 3.8% [29]. In the case of osteoarthritis of hip, prevalence on general population has been observed between 0.9 and 7.4% [28, 29]. Studies exploring chronic shoulder pain in population are few and use definition not comparable with our report [30]. The prevalence of fibromyalgia obtained in our study was slightly higher than the pooled prevalence informed in a last available systematic review [33], although our number is included in the heterogeneity of estimations. However, one limitation relates to the questionnaire used to detect fibromyalgia, which was not specifically designed for this disorder. Moreover, the items that we selected to fulfil the diagnostic criteria have not been validated for this purpose.

In this study we compared the impact of musculoskeletal disorders using two different approaches. The first one was the calculus of DALYs, which is a traditional way to estimate the burden of diseases [3] and in the case of this study combines the prevalence of musculoskeletal disorders and disability weights for their sequels. However, DALYs carry some inconveniences. First, it uses disability weights obtained from surveys among experts which results are combined with studies carried out in countries that not necessary represent the reality of the country under study [2, 34, 35]. Second, in order to estimate the relative magnitude of the burden for one disease, it is necessary to estimate the burden of an exhaustive list of other diseases, which exceed three hundred entities to date [1]. Furthermore, authors have pointed out that the current procedure used to calculate DALYs requires a high level of technical sophistication [20] that precludes traditional government agencies from replicating results obtained by the leading institutions that have performed the latest burden of disease studies [7].

Beyond these considerations, the last burden of disease study performed by the Institute of Health Metric and Evaluation (IHME) estimated for Chile, year 2017, a burden for all musculoskeletal disorders equal to 544.557 DALYs [396.781–724.208] [1], which is no far from our estimate for the broad disorder of chronic musculoskeletal pain disorder. The IHME also reported a burden for all osteoarthritis equal to 34.086 DALYs [17.011–67.496], which does not differ significantly from our added estimates for osteoarthritis of hip and knee. However, we found disagreement in the magnitude of low back pain, since the IHME predicts more than twice the burden we estimated in our study. This disparity could be due to differences in the definition of the disorder, because we only considered chronic low back pain, whereas the IHME also included the acute one [36]. Finally, we found a significant burden for fibromyalgia which overpasses the burden for chronic low back pain; and also, for chronic shoulder pain, which overpasses the burden for osteoarthritis of hip and knee. Unfortunately, fibromyalgia and chronic shoulder pain are not studied as separate entities by the IHME.

Our second approach used a more innovative way to estimate the impact of diseases, in this case combining the prevalence of disorders and the LHSU attributable to them. Health state utilities, as was mentioned previously, captures the preference of society about different health states, which makes it different from the concept of disability used in DALY construction [8, 37]. HSU are also used as an input to estimate the quality adjusted life years (QALY) a metric often employed in cost-effective analysis for the assessment of health technologies [37]. Furthermore, the population LHSU that we are presenting in Table 4, can be also interpreted in some extent as ‘prevalent – QALYs lost’ attributable to each disorder (valuable information for economic evaluation on musculoskeletal disorder is provided in the supplementary material).

In comparison with DALY, this procedure has at least five advantages: firstly, the prevalence and the LHSU can be extracted from a single source (i.e. a national representative survey); secondly, the procedure is easily implementable since it is based on a simple regression model; thirdly, provides valuable information at individual level (i.e. average LHSU) and at population level (i.e. total LHSU); fourthly, because the procedure is based on a simple regression model, it is easy to generate estimates adjusted and unadjusted by comorbidities and other covariables; and finally, the procedure allows us to estimate a relative impact of each disease without including an extended list of other diseases in the model.

Results obtained through this procedure remarks how the magnitude of LSHU at individual level drops after adjustment by comorbidity and covariables in several of the musculoskeletal disorders. For instance, in the case of osteoarthritis of hip and knee their LHSU fall more than a half, from approximately twenty-seven to around ten LSHU, or in the case of chronic shoulder pain which LSHU after adjustment fall to almost zero LSHU. Also, it is noticeably that results of total LSHU at population level are not coincident with those obtained using DALY. Although both procedures highlight the relevance of fibromyalgia and chronic low back pain over the other disorders, the results do not show proportionality between their magnitudes. The most extreme difference is observed in the case of chronic shoulder pain. This shows that, in the assessment of the impact of diseases, a metric based in disability or social preferences of health states (i.e., LHSU) are not necessarily interchangeable.

Finally, pain and discomfort as a domain of LHSU were associated roughly with a half of total LHSU in people with different musculoskeletal disorders. However, this analysis performed on general population, showed that this domain was associated to a 53,6% [51.2–56.1] of total LHSU, showing that the pain and discomfort are not exclusive from musculoskeletal disorders, and the magnitude could be attributed to metric characteristics of the instrument.

This study has several limitations. Firstly, we used a sub-sample from the original sample of the survey which could bias our estimates. Although our missing data analysis shows that the population with and without missing variables could be comparable, that analysis do not discard completely selection bias. In addition, although the ChNHS explored around twenty diseases potentially able to be included in the models for adjusting results, to analyses and edit each one was beyond our resources and the scope of this study, reason why we selected only three prevalent health conditions (i.e., hypertension, diabetes and depression) usually associated to musculoskeletal disorders [16, 38]. The inclusion of more diseases to adjust results could be conducted to smaller magnitude of DALY or LHSU.

## Conclusions

This study provides relevant information about musculoskeletal disorders combining different approaches. Our results suggest that chronic musculoskeletal pain disorder is a major source of burden and LHSU. Fibromyalgia is highly likely to be associated to a high burden of disease as well, nevertheless, we faced limitations that should be addressed in future studies. Using LHSU assessing the impact of musculoskeletal disorders offers a straightforward and productive opportunity to deepen the knowledge about these conditions.

## Supplementary Information

**Additional file 1 Table S1**. Disability weights, labels, description by sequel†, and level of pain used to classify each disorder in each sequel (adapted from Salomon JA, et al. The Lancet Global Health 2015, 3(11):e712–723). **Table S2**. Description of the sample stratified for musculoskeletal conditions. Chilean National Health Survey 2016–2017 (*n* = 5077). **Figure S1**. Smoothed prevalence of selected musculoskeletal disorder by age. Chilean National Health Survey 2016–2017 (*n* = 5077). **Table S3**. Bivariate and Multivariate regression models for Loss of Heath State Utilities† using data from the Chilean National Health Survey 2016–2017 (n = 5077). **Table S4**. Rate of disability adjusted life years and loss of health state utilities†, per 100,000 inhabitants for musculoskeletal disorders, using data from the Chilean National Health Survey 2016–2017 (*n* = 5077). **Table S5**. Smoothed prevalence of sequels of musculoskeletal disorders, using data from the Chilean National Health Survey 2016–2017 (*n* = 5077). **Table S6**. Disability Adjusted Life Years for selected musculoskeletal disorders for Chile, 2017. **Table S7**. Fraction of loss of health state utilities attributable to domains of the EQ. 5D questionnaire for general population and selected musculoskeletal disorders, using data from the Chilean National Health Survey 2016–2017 (n = 5077). **Table S8**. Health state utilities attributable, in people with and without selected musculoskeletal conditions, using data from the Chilean National Health Survey 2016–2017 (n = 5077).

## Data Availability

The data from the National Health Survey is publicly available under request to the Ministry of Health of Chile (www.minsal.cl).

## Abbreviations

COPCOPRD: Community Oriented Programme for the Control of Rheumatic Disease Core Questionnaire
CIDI-SF: Composite International Diagnosis Interview short-form
COMO: Microsiumulation process for Comorbidity Correction
ChNHS: Chilean National Health Survey
DALY: Disability Adjusted Life Years
DSM-IV: Diagnostic and Statistical Manual of Mental Disorders 4th edition
EQ 5 D: EuroQol 5 Dimensions
HSU: Health State Utility
IHME: Institute of Health Metric and Evaluation
LHSU: Loss of Health State Utilities
QALY: Quality Adjusted Life Years
YLD: Years Lived with Disability
YLL: Years of Life Lost by premature death
