# Supplementary material for: Prevalence, burden of disease, and lost in health state utilities attributable to chronic musculoskeletal disorders and pain in Chile
Source: BMC Public Health. 2021 May 17;21:937. doi: 10.1186/s12889-021-10953-z (PMC8130395; doi:10.1186/s12889-021-10953-z)
Supplement: Supplementary file 1 — Additional file 1 Table S1. Disability weights, labels, description by sequel†, and level of pain used to classify each disorder in each sequel (adapted from Salomon JA, et al. The Lancet Global Health 2015, 3(11):e712–723). Table S2. Description of the sample stratified for musculoskeletal conditions. Chilean National Health Survey 2016–2017 (n = 5077). Figure S1. Smoothed prevalence of selected musculoskeletal disorder by age. Chilean National Health Survey 2016–2017 (n = 5077). Table S3. Bivariate and Multivariate regression models for Loss of Heath State Utilities† using data from the Chilean National Health Survey 2016–2017 (n = 5077). Table S4. Rate of disability adjusted life years and loss of health state utilities†, per 100,000 inhabitants for musculoskeletal disorders, using data from the Chilean National Health Survey 2016–2017 (n = 5077). Table S5. Smoothed prevalence of sequels of musculoskeletal disorders, using data from the Chilean National Health Survey 2016–2017 (n = 5077). Table S6. Disability Adjusted Life Years for selected musculoskeletal disorders for Chile, 2017. Table S7. Fraction of loss of health state utilities attributable to domains of the EQ. 5D questionnaire for general population and selected musculoskeletal disorders, using data from the Chilean National Health Survey 2016–2017 (n = 5077). Table S8. Health state utilities attributable, in people with and without selected musculoskeletal conditions, using data from the Chilean National Health Survey 2016–2017 (n = 5077). [file 12889_2021_10953_MOESM1_ESM.docx]

**Supplement Material**

**The Prevalence, Burden of Disease, and Lost in Health State Utilities attributable to Chronic Musculoskeletal and Pain in Chile**

Pedro Zitko; Norberto Bilbeny; Carlos Balmaceda; Tomas Abbott; Cesar Carcamo; Manuel Espinoza

Index

- Table S1. Disability weights, labels, description by sequel,

and level of pain used to classify each disorder in each sequel. 2

- Table S2. Description of the sample stratified for musculoskeletal conditions. 3
- Figure S1. Smoothed prevalence of selected musculoskeletal disorder by age. 4
- Table S3. Bivariate and Multivariate regression models for Loss of Heath State Utilities 5
- Table S4. Rate of disability adjusted life years and Loss of Health State Utilities

per 100,000 inhabitants for musculoskeletal disorders. 6

- Table S5. Smoothed prevalence of sequels of musculoskeletal disorders. 7
- Table S6. Disability Adjusted Life Years for selected musculoskeletal disorders for Chile 13
- Table S7. Fraction of loss of health state utilities attributable to domains of the EQ5D

questionnaire for general population and selected musculoskeletal disorders. 16

- Table S8. Health state utilities attributable, in people with and without selected musculoskeletal

conditions. 17

|  |  |  |  |  |  |  |  |
| --- | --- | --- | --- | --- | --- | --- | --- |
| **Disorder** | **Sequel of the disorder** | **Pain 0-10** | **Disabiliy weight** | **LCI** | **UCI** | **Label of disability weight** | **Description** |
| Low back | mild | 1-3 | 0.020 | 0.011 | 0.035 | Low back pain, mild | Has mild back pain, which causes some difficulty dressing, standing, and lifting things. |
| Low back | moderate | 4-6 | 0.054 | 0.035 | 0.079 | Low back pain, moderate | Has moderate back pain, which causes difficulty dressing, sitting, standing, walking, and lifting things. |
| Low back | severe | 7-8 | 0.272 | 0.182 | 0.373 | Back pain, severe, without leg pain | Has severe back pain, which causes difficulty dressing, sitting, standing, walking, and lifting things. The person sleeps poorly and feels worried. |
| Low back | most severe | 9-10 | 0.372 | 0.250 | 0.506 | Back pain, most severe, without leg pain | Has constant back pain, which causes difficulty dressing, sitting, standing, walking, and lifting things. The person sleeps poorly, is worried, and has lost some enjoyment in life. |
| Knee | mild | 1-3 | 0.023 | 0.013 | 0.037 | Musculoskeletal problems, lower limbs, mild | Has pain in the leg, which causes some difficulty running, walking long distances, and getting up and down. |
| Knee | moderate | 4-6 | 0.079 | 0.054 | 0.110 | Musculoskeletal problems, lower limbs, moderate | Has moderate pain in the leg, which makes the person limp, and causes some difficulty walking, standing, lifting and carrying heavy things, getting up and down and sleeping. |
| Knee | severe | 7-10 | 0.165 | 0.112 | 0.232 | Musculoskeletal problems, lower limbs, severe | Has severe pain in the leg, which makes the person limp and causes a lot of difficulty walking, standing, lifting and carrying heavy things, getting up and down, and sleeping. |
| Hip | mild | 1-3 | 0.023 | 0.013 | 0.037 | Musculoskeletal problems, lower limbs, mild | Has pain in the leg, which causes some difficulty running, walking long distances, and getting up and down. |
| Hip | moderate | 4-6 | 0.079 | 0.054 | 0.110 | Musculoskeletal problems, lower limbs, moderate | has moderate pain in the leg, which makes the person limp, and causes some difficulty walking, standing, lifting and carrying heavy things, getting up and down and sleeping. |
| Hip | severe | 7-10 | 0.165 | 0.112 | 0.232 | Musculoskeletal problems, lower limbs, severe | Has severe pain in the leg, which makes the person limp and causes a lot of difficulty walking, standing, lifting and carrying heavy things, getting up and down, and sleeping. |
| Shoulder | mild | 1-3 | 0.028 | 0.017 | 0.045 | Musculoskeletal problems, upper limbs, mild | Has mild pain and stiffness in the arms and hands. The person has some difficulty lifting, carrying and holding things. |
| Shoulder | moderate -severe | 4-10 | 0.117 | 0.080 | 0.163 | Musculoskeletal problems, upper limbs, moderate | Has moderate pain and stiffness in the arms and hands, which causes difficulty lifting, carrying, and holding things, and trouble sleeping because of the pain. |
| Fibromialgia | mild | - | - | - | - | - | - |
| Fibromialgia | moderate | 4-6 | 0.317 | 0.216 | 0.440 | Musculoskeletal problems, generalized, moderate | Has pain and deformity in most joints, causing difficulty moving around, getting up and down, and using the hands for lifting and carrying. The person often feels fatigue. |
| Fibromialgia | severe | 7-10 | 0.581 | 0.403 | 0.739 | Musculoskeletal problems, generalized, severe | Has severe, constant pain and deformity in most joints, causing difficulty moving around, getting up and down, eating, dressing, lifting, carrying and using the hands. The person often feels sadness, anxiety and extreme fatigue. |
| Chronic MSK pain | mild | - | 0.020 | 0.011 | 0.035 | Low back pain, mild | |
| Chronic MSK pain | moderate | 4-6 | 0.054 | 0.035 | 0.079 | Low back pain, moderate | |
| Chronic MSK pain | severe | 7-10 | 0.272 | 0.182 | 0.373 | Back pain, severe, without leg pain | |

**Table S1**. Disability weights, labels, description by sequel†, and level of pain used to classify each disorder in each sequel (adapted from Salomon JA, et al. The Lancet Global Health 2015, 3(11):e712-723)

**Table S2**. Description of the sample stratified for musculoskeletal conditions. Chilean National Health Survey 2016-2017 (n=5,077)

|  | Chronic low back pain (n = 241) | |  | Chronic shoulder pain (n = 170) | |  | Osteoarthritis of Hip  (n = 164) | |  | Osteoarthritis of Knee (n = 242) | |  | | Fibromyalgia  (n = 225) | | | |  | | Chronic Musculosqueletal pain (n = 1.167) | |
| --- | --- | --- | --- | --- | --- | --- | --- | --- | --- | --- | --- | --- | --- | --- | --- | --- | --- | --- | --- | --- | --- |
|  | **mean** | **CI** |  | **mean** | **CI** |  | **mean** | **CI** |  | **mean** | **CI** | |  | | **mean** | **CI** |  | | **mean** | | **CI** |
| Health state (mean) | 30.4 | [ 26-34.9 ] |  | 31.9 | [ 25.1-38.6 ] |  | 49.7 | [ 41.5-58 ] |  | 48.5 | [ 41.6-55.5 ] | |  | | 58.2 | [ 51.8-64.6 ] |  | | 35.0 | | [ 32.5-37.5 ] |
| Age (mean) | 43.1 | [ 39.5-46.8 ] |  | 54.6 | [ 51.5-57.7 ] |  | 63.1 | [ 59.1-67 ] |  | 61.7 | [ 59.1-64.4 ] | |  | | 54.8 | [ 49.3-60.4 ] |  | | 49.7 | | [ 47.9-51.5 ] |
|  |  |  |  |  |  |  |  |  |  |  |  | |  | |  |  |  | |  | |  |
|  | **%** | **CI** |  | **%** | **CI** |  | **%** | **CI** |  | **%** | **CI** | |  | | **%** | **CI** |  | | **%** | | **CI** |
| Sex (females) | 52.4 | [ 41.1-63.8 ] |  | 67.5 | [ 55.2-79.8 ] |  | 90.6 | [ 84.5-96.7 ] |  | 78.4 | [ 66.8-90.0 ] | |  | | 82.8 | [ 72.4-93.2 ] |  | | 66.4 | | [ 61.2-71.6 ] |
| Marita status | |  |  |  |  |  |  |  |  |  |  | |  | |  |  |  | |  | |  |
| *married/ cohabiting* | 62.2 | [ 51.7-72.8 ] |  | 68.4 | [ 57.5-79.3 ] |  | 51.6 | [ 39.4-63.8 ] |  | 57.7 | [ 45.5-69.8 ] | |  | | 54.9 | [ 43.8-66.1 ] |  | | 60.3 | | [ 55.3-65.3 ] |
| *annulled/separated/divorced* | 10.0 | [ 3.0-17.0 ] |  | 9.7 | [ 3.5-16.0 ] |  | 15.1 | [ 5.6-24.6 ] |  | 17.9 | [ 6.2-29.7 ] | |  | | 8.3 | [ 3.7-13.0 ] |  | | 10.5 | | [ 7.6-13.4 ] |
| *widower* | 3.1 | [ 1-5.2.0 ] |  | 8.5 | [ 2.7-14.3 ] |  | 19.7 | [ 10.8-28.6 ] |  | 14.0 | [ 7.1-20.9 ] | |  | | 11.7 | [ 6.1-17.2 ] |  | | 5.6 | | [ 4.2-7.0 ] |
| *Single* | 24.6 | [ 15.6-33.6 ] |  | 13.3 | [ 6.0-20.6 ] |  | 13.5 | [ 4.0-23.0 ] |  | 10.4 | [ 3.8-17.0 ] | |  | | 25.0 | [ 14.6-35.5 ] |  | | 23.6 | | [ 19.1-28.1 ] |
| Education |  |  |  |  |  |  |  |  |  |  |  | |  | |  |  |  | |  | |  |
| *> 12 years* | 27.3 | [ 17.3-37.3 ] |  | 11.2 | [ 3.6-18.8 ] |  | 12.3 | [ 1.3-23.3 ] |  | 11.8 | [ 3.8-19.8 ] | |  | | 18.6 | [ 9.1-28.2 ] |  | | 16.0 | | [ 12.2-19.8 ] |
| *9 - 12 years* | 46.0 | [ 34.6-57.3 ] |  | 44.9 | [ 31.7-58.2 ] |  | 27.3 | [ 16.9-37.6 ] |  | 33.7 | [ 21.6-45.7 ] | |  | | 38.0 | [ 27.1-48.9 ] |  | | 50.3 | | [ 45.1-55.4 ] |
| *< 9 years* | 26.7 | [ 17.7-35.7 ] |  | 43.8 | [ 30.6-57.1 ] |  | 60.4 | [ 48.0-72.8 ] |  | 54.5 | [ 42.3-66.8 ] | |  | | 43.4 | [ 32.3-54.5 ] |  | | 33.7 | | [ 29.1-38.3 ] |
| Ocupation |  |  |  |  |  |  |  |  |  |  |  | |  | |  |  |  | |  | |  |
| *working for salary* | 54.7 | [ 43.7-65.7 ] |  | 46.8 | [ 33.5-60.1 ] |  | 20.2 | [ 9.8-30.6 ] |  | 23.3 | [ 14.2-32.4 ] | |  | | 41.7 | [ 30.2-53.2 ] |  | | 49.3 | | [ 44.1-54.5 ] |
| *loking for work* | 0.9 | [ -0.2-2.0 ] |  | 0.3 | [ -0.1-0.8 ] |  | 0.1 | [ -0.1-0.3 ] |  | 2.8 | [ -0.9-6.5 ] | |  | | 2.0 | [ 0.3-3.7 ] |  | | 2.6 | | [ 1.3-3.9 ] |
| *working without salary* | 15.3 | [ 8.5-22.0 ] |  | 31.4 | [ 17.6-45.2 ] |  | 32.2 | [ 20.5-43.9 ] |  | 24.6 | [ 15.6-33.7 ] | |  | | 23.5 | [ 15.0-32.0 ] |  | | 23.7 | | [ 19.6-27.8 ] |
| *Not working, and not looking for* | 29.2 | [ 19-39.3 ] |  | 21.4 | [ 13.5-29.3 ] |  | 47.5 | [ 35.5-59.6 ] |  | 49.2 | [ 37.1-61.4 ] | |  | | 32.7 | [ 22.1-43.4 ] |  | | 24.4 | | [ 20.2-28.6 ] |
| Comorbidity |  |  |  |  |  |  |  |  |  |  |  | |  | |  |  |  | |  | |  |
| *Hypertension* | 31.4 | [ 21.0-41.9 ] |  | 53.8 | [ 40.9-66.8 ] |  | 62.4 | [ 49.7-75.1 ] |  | 59.7 | [ 47.4-72.0 ] | |  | | 54.6 | [ 43.3-65.9 ] |  | | 38.3 | | [ 33.3-43.2 ] |
| *Diabetes* | 9.9 | [ 3.7-16.0 ] |  | 13.6 | [ 7.4-19.7 ] |  | 26.7 | [ 16.8-36.5 ] |  | 37.3 | [ 25.4-49.3 ] | |  | | 24.4 | [ 15.1-33.7 ] |  | | 18.8 | | [ 15.0-22.6 ] |
| *Depresive episode* | 25.3 | [ 15.3-35.3 ] |  | 29.7 | [ 15.4-44.1 ] |  | 23.8 | [ 13.1-34.6 ] |  | 28.3 | [ 16.0-40.6 ] | |  | | 56.9 | [ 45.4-68.4 ] |  | | 27.5 | | [ 22.6-32.4 ] |

*CI: confidence intervals 95%*

**Figure S1**. Smoothed prevalence of selected musculoskeletal disorder by age. Chilean National Health Survey 2016-2017 (n=5,077)

**General population older than 14 years**

**Men Women**

**Table S3**. Bivariate and Multivariate regression models for Loss of Heath State Utilities† using data from the Chilean National Health Survey 2016-2017 (n=5,077)

|  | Bivariate | |  | Multivariate 1 | |  | Multivariate 2 | |
| --- | --- | --- | --- | --- | --- | --- | --- | --- |
|  | HSU | CI |  | HSU | CI |  | HSU | CI |
| Intercept | - | - |  | 1.2 | [ -2.7 to 5.1 ] |  | 0.3 | [ -3.6 to 4.1 ] |
| Age (each 10 years) | 4.2 | [ 3.6 to 4.7 ] |  | 2.4 | [ 1.7 to 3.2 ] |  | 2.5 | [ 1.7 to 3.2 ] |
| Sex (females) | 7.4 | [ 5.3 to 9.6 ] |  | 2.0 | [ -0.1 to 4.0 ] |  | 2.3 | [ 0.2 to 4.4 ] |
| Marita status | |  |  |  |  |  |  |  |
| *married/ cohabiting* | 0.0 | - |  | 0.0 | - |  | 0.0 | - |
| *annulled/separated/divorced* | 5.8 | [ 1.2 to 10.4 ] |  | 1.5 | [ -2.7 to 5.8 ] |  | 1.4 | [ -3.0 to 5.8 ] |
| *widower* | 16.1 | [ 10.5 to 21.6 ] |  | 2.6 | [ -2.4 to 7.7 ] |  | 3.7 | [ -1.5 to 8.8 ] |
| *Single* | -6.3 | [ -8.5 to -4.2 ] |  | -1.3 | [ -3.5 to 1.0 ] |  | -1.2 | [ -3.6 to 1.2 ] |
| Education |  |  |  |  |  |  |  |  |
| *> 12 years* | 0.0 | - |  | 0.0 | - |  | 0.0 | - |
| *9 - 12 years* | 4.6 | [ 2.1 to 7.1 ] |  | 3.5 | [ 1.4 to 5.7 ] |  | 2.7 | [ 0.5 to 4.9 ] |
| *< 9 years* | 15.6 | [ 12.8 to 18.5 ] |  | 5.7 | [ 3.0 to 8.4 ] |  | 5.4 | [ 2.6 to 8.2 ] |
| Ocupation |  |  |  |  |  |  |  |  |
| *working for salary* | 0.0 | - |  | 0.0 | - |  | 0.0 | - |
| *loking for work* | -2.0 | [ -6.5 to 2.6 ] |  | 0.3 | [ -4.0 to 4.6 ] |  | 0.3 | [ -4.0 to 4.6 ] |
| *working without salary* | 7.5 | [ 4.5 to 10.5 ] |  | 2.1 | [ -0.8 to 5 ] |  | 1.9 | [ -1.2 to 5.0 ] |
| *Not working, and not looking for* | 8.1 | [ 5.3 to 10.8 ] |  | 5.5 | [ 3.2 to 7.8 ] |  | 6.4 | [ 4.0 to 8.8 ] |
|  |  |  |  |  |  |  |  |  |
| Hypertension | 12.3 | [ 9.8 to 14.8 ] |  | 0.6 | [ -2.2 to 3.5 ] |  | 1.3 | [ -1.7 to 4.3 ] |
| Diabetes | 13.7 | [ 9.7 to 17.6 ] |  | 5.0 | [ 1.7 to 8.4 ] |  | 5.2 | [ 1.6 to 8.8 ] |
| Depresive episode | 17.5 | [ 13.9 to 21 ] |  | 14.0 | [ 10.8 to 17.3 ] |  | 15.5 | [ 12.3 to 18.8 ] |
| Chronic low back pain | 8.6 | [ 4 to 13.2 ] |  | 5.5 | [ 1.9 to 9.1 ] |  | - | - |
| Chronic shoulder pain | 9.7 | [ 2.9 to 16.5 ] |  | 0.5 | [ -6.2 to 7.2 ] |  | - | - |
| Osteoarthritis of Hip | 27.9 | [ 19.6 to 36.2 ] |  | 7.4 | [ -0.4 to 15.1 ] |  | - | - |
| Osteoarthritis of Knee | 27.0 | [ 20 to 34.1 ] |  | 10.1 | [ 2.5 to 17.7 ] |  | - | - |
| Fibromyalgia | 37.3 | [ 30.8 to 43.7 ] |  | 23.2 | [ 16.7 to 29.7 ] |  | - | - |
| Chronic Musculosqueletal pain | 16.0 | [ 13.3 to 18.8 ] |  | - | - |  | 9.9 | [ 7.4 to 12.5 ] |

*CI: confidence intervals 95%*

*HSU: Health State Utilities*

*Loss of HSU are anchored in values 0 and 100, equivalent to perfect health and death, respectively.*

**Table S4**. Rate of disability adjusted life years and loss of health state utilities†, per 100,000 inhabitants for musculoskeletal disorders, using data from the Chilean National Health Survey 2016-2017 (n=5,077)

|  | rate of DALY | CI |  | rate of HSU lost | CI | |  |
| --- | --- | --- | --- | --- | --- | --- | --- |
| Chronic low back pain | 775.2 | [ 768.0 - 782.4 ] |  | 244.8 | | [ 199.3 - 290 ] | |
| Chronic shoulder pain | 225.7 | [ 223.3 - 228.1 ] |  | 9.1 | | [ 7.1 - 11.1 ] | |
| Osteoarthritis of Hip | 54.3 | [ 52.4 - 56.2 ] |  | 99.5 | | [ 80.7 - 118.2 ] | |
| Osteoarthritis of Knee | 88.6 | [ 86.2 - 90.9 ] |  | 226.4 | | [ 180.3 - 272.6 ] | |
| Fibromyalgia | 1.299.0 | [ 1292.2 - 1305.9 ] |  | 594.1 | | [ 483.6 - 702.5 ] | |
| Chronic Musculosketal pain | 2.735.8 | [ 2726.9 - 2744.6 ] |  | 1.410.0 | | [ 1290.7 - 1529.5 ] | |

*CI: confidence intervals 95% / DALY: disability adjusted life years / HSU: Health State Utilities*

*† Loss of HSU are anchored in values 0 and 1, equivalent to perfect health and death, respectively.*

**Table S5**. Smoothed prevalence of sequels of musculoskeletal disorders, using data from the Chilean National Health Survey 2016-2017 (n=5,077)

Chronic low back pain mild

|  | Men | | | Women | | | Total | | |
| --- | --- | --- | --- | --- | --- | --- | --- | --- | --- |
|  | **%** | **LCI** | **UCI** | **%** | **LCI** | **UCI** | **%** | **LCI** | **UCI** |
| 0-9 | 0.0 | 0.0 | 0.0 | 0.0 | 0.0 | 0.0 | 0.0 | 0.0 | 0.0 |
| 10-19 | 1.0 | 0.5 | 1.7 | 0.1 | 0.0 | 0.2 | 0.6 | 0.3 | 0.9 |
| 20-29 | 1.6 | 1.1 | 2.2 | 0.2 | 0.1 | 0.3 | 0.9 | 0.6 | 1.2 |
| 30-39 | 1.2 | 0.9 | 1.6 | 0.1 | 0.1 | 0.2 | 0.7 | 0.5 | 0.9 |
| 40-49 | 0.9 | 0.6 | 1.2 | 0.1 | 0.0 | 0.2 | 0.5 | 0.4 | 0.7 |
| 50-59 | 0.7 | 0.4 | 1.0 | 0.1 | 0.0 | 0.1 | 0.4 | 0.2 | 0.5 |
| 60-69 | 0.5 | 0.3 | 0.8 | 0.1 | 0.0 | 0.1 | 0.3 | 0.2 | 0.4 |
| 70-79 | 0.4 | 0.2 | 0.7 | 0.0 | 0.0 | 0.1 | 0.2 | 0.1 | 0.3 |
| 80+ | 0.3 | 0.1 | 0.5 | 0.0 | 0.0 | 0.1 | 0.1 | 0.1 | 0.2 |
| Total | 0.9 | 0.7 | 1.0 | 0.1 | 0.1 | 0.1 | 0.5 | 0.4 | 0.6 |

Chronic low back pain moderate

|  | Men | | | Women | | | Total | | |
| --- | --- | --- | --- | --- | --- | --- | --- | --- | --- |
|  | **%** | **LCI** | **UCI** | **%** | **LCI** | **UCI** | **%** | **LCI** | **UCI** |
| 0-9 | 0.0 | 0.0 | 0.0 | 0.0 | 0.0 | 0.0 | 0.0 | 0.0 | 0.0 |
| 10-19 | 2.4 | 1.8 | 3.0 | 1.8 | 1.3 | 2.5 | 2.1 | 1.7 | 2.6 |
| 20-29 | 4.4 | 3.8 | 5.1 | 3.4 | 2.8 | 4.1 | 3.9 | 3.4 | 4.4 |
| 30-39 | 4.2 | 3.6 | 4.8 | 3.2 | 2.7 | 3.8 | 3.7 | 3.3 | 4.1 |
| 40-49 | 4.0 | 3.3 | 4.6 | 3.1 | 2.6 | 3.5 | 3.5 | 3.1 | 3.9 |
| 50-59 | 3.8 | 3.1 | 4.5 | 2.9 | 2.5 | 3.3 | 3.3 | 2.9 | 3.7 |
| 60-69 | 3.6 | 2.8 | 4.4 | 2.7 | 2.4 | 3.1 | 3.1 | 2.7 | 3.6 |
| 70-79 | 3.4 | 2.5 | 4.4 | 2.6 | 2.2 | 3.1 | 2.9 | 2.5 | 3.4 |
| 80+ | 3.2 | 2.3 | 4.2 | 2.4 | 2.0 | 2.9 | 2.7 | 2.3 | 3.2 |
| Total | 3.2 | 3.0 | 3.4 | 2.7 | 2.5 | 2.9 | 2.8 | 2.7 | 3.0 |

*LCI: Lower confidence Interval 95% / UCI: Upper confidence Interval 95%*

Chronic low back pain severe

|  | Men | | | Women | | | Total | | |
| --- | --- | --- | --- | --- | --- | --- | --- | --- | --- |
|  | **%** | **LCI** | **UCI** | **%** | **LCI** | **UCI** | **%** | **LCI** | **UCI** |
| 0-9 | 0.0 | 0.0 | 0.0 | 0.0 | 0.0 | 0.0 | 0.0 | 0.0 | 0.0 |
| 10-19 | 0.5 | 0.3 | 0.7 | 0.8 | 0.6 | 1.1 | 0.6 | 0.5 | 0.8 |
| 20-29 | 1.0 | 0.8 | 1.3 | 1.7 | 1.4 | 2.1 | 1.4 | 1.2 | 1.6 |
| 30-39 | 1.2 | 0.9 | 1.4 | 1.9 | 1.6 | 2.3 | 1.6 | 1.4 | 1.8 |
| 40-49 | 1.3 | 1.1 | 1.6 | 2.2 | 1.9 | 2.5 | 1.8 | 1.6 | 2.0 |
| 50-59 | 1.5 | 1.2 | 1.8 | 2.5 | 2.1 | 2.9 | 2.0 | 1.8 | 2.3 |
| 60-69 | 1.7 | 1.3 | 2.1 | 2.8 | 2.4 | 3.3 | 2.3 | 2.0 | 2.6 |
| 70-79 | 1.9 | 1.5 | 2.4 | 3.2 | 2.6 | 3.8 | 2.6 | 2.3 | 3.0 |
| 80+ | 2.2 | 1.7 | 2.8 | 3.7 | 3.0 | 4.3 | 3.2 | 2.7 | 3.6 |
| Total | 1.0 | 1.0 | 1.1 | 2.0 | 1.8 | 2.1 | 1.4 | 1.4 | 1.5 |

Chronic low back pain hyper-severe

|  | Men | | | Women | | | Total | | |
| --- | --- | --- | --- | --- | --- | --- | --- | --- | --- |
|  | **%** | **LCI** | **UCI** | **%** | **LCI** | **UCI** | **%** | **LCI** | **UCI** |
| 0-9 | 0.0 | 0.0 | 0.0 | 0.0 | 0.0 | 0.0 | 0.0 | 0.0 | 0.0 |
| 10-19 | 0.2 | 0.1 | 0.2 | 0.7 | 0.4 | 1.0 | 0.4 | 0.3 | 0.6 |
| 20-29 | 0.3 | 0.2 | 0.4 | 1.3 | 1.0 | 1.7 | 0.8 | 0.6 | 1.0 |
| 30-39 | 0.3 | 0.3 | 0.4 | 1.4 | 1.1 | 1.8 | 0.9 | 0.7 | 1.0 |
| 40-49 | 0.3 | 0.3 | 0.4 | 1.5 | 1.3 | 1.8 | 0.9 | 0.8 | 1.1 |
| 50-59 | 0.4 | 0.3 | 0.5 | 1.6 | 1.4 | 1.9 | 1.0 | 0.9 | 1.2 |
| 60-69 | 0.4 | 0.3 | 0.5 | 1.7 | 1.4 | 2.0 | 1.1 | 0.9 | 1.3 |
| 70-79 | 0.4 | 0.3 | 0.5 | 1.8 | 1.5 | 2.2 | 1.2 | 1.0 | 1.4 |
| 80+ | 0.4 | 0.3 | 0.6 | 2.0 | 1.6 | 2.4 | 1.4 | 1.2 | 1.7 |
| Total | 0.3 | 0.2 | 0.3 | 1.3 | 1.2 | 1.5 | 0.8 | 0.7 | 0.8 |

Chronic Shoulder pain mild

|  | Men | | | Women | | | Total | | |
| --- | --- | --- | --- | --- | --- | --- | --- | --- | --- |
|  | **%** | **LCI** | **UCI** | **%** | **LCI** | **UCI** | **%** | **LCI** | **UCI** |
| 0-9 | 0.0 | 0.0 | 0.0 | 0.0 | 0.0 | 0.0 | 0.0 | 0.0 | 0.0 |
| 10-19 | 0.0 | 0.0 | 0.1 | 0.0 | 0.0 | 0.0 | 0.0 | 0.0 | 0.1 |
| 20-29 | 0.1 | 0.0 | 0.1 | 0.1 | 0.0 | 0.1 | 0.1 | 0.0 | 0.1 |
| 30-39 | 0.1 | 0.0 | 0.1 | 0.1 | 0.1 | 0.1 | 0.1 | 0.1 | 0.1 |
| 40-49 | 0.1 | 0.1 | 0.1 | 0.1 | 0.1 | 0.1 | 0.1 | 0.1 | 0.1 |
| 50-59 | 0.1 | 0.1 | 0.2 | 0.1 | 0.1 | 0.1 | 0.1 | 0.1 | 0.1 |
| 60-69 | 0.1 | 0.1 | 0.2 | 0.1 | 0.1 | 0.2 | 0.1 | 0.1 | 0.2 |
| 70-79 | 0.2 | 0.1 | 0.2 | 0.2 | 0.1 | 0.3 | 0.2 | 0.1 | 0.2 |
| 80+ | 0.2 | 0.1 | 0.3 | 0.2 | 0.1 | 0.3 | 0.2 | 0.1 | 0.3 |
| Total | 0.1 | 0.1 | 0.1 | 0.1 | 0.1 | 0.1 | 0.1 | 0.1 | 0.1 |

*LCI: Lower confidence Interval 95% / UCI: Upper confidence Interval 95%*

Chronic shoulder pain moderate and severe

|  | Men | | | Women | | | Total | | |
| --- | --- | --- | --- | --- | --- | --- | --- | --- | --- |
|  | **%** | **LCI** | **UCI** | **%** | **LCI** | **UCI** | **%** | **LCI** | **UCI** |
| 0-9 | 0.0 | 0.0 | 0.0 | 0.0 | 0.0 | 0.0 | 0.0 | 0.0 | 0.0 |
| 10-19 | 0.3 | 0.3 | 0.4 | 0.6 | 0.5 | 0.8 | 0.5 | 0.4 | 0.6 |
| 20-29 | 0.8 | 0.7 | 0.9 | 1.6 | 1.3 | 1.8 | 1.2 | 1.0 | 1.3 |
| 30-39 | 1.1 | 1.0 | 1.3 | 2.2 | 1.9 | 2.5 | 1.6 | 1.5 | 1.8 |
| 40-49 | 1.6 | 1.3 | 1.8 | 3.0 | 2.7 | 3.4 | 2.3 | 2.1 | 2.5 |
| 50-59 | 2.2 | 1.9 | 2.5 | 4.2 | 3.7 | 4.6 | 3.2 | 2.9 | 3.5 |
| 60-69 | 3.0 | 2.5 | 3.5 | 5.7 | 5.2 | 6.3 | 4.4 | 4.1 | 4.8 |
| 70-79 | 4.1 | 3.4 | 4.8 | 7.8 | 7.0 | 8.6 | 6.2 | 5.7 | 6.7 |
| 80+ | 5.9 | 5.0 | 6.9 | 11.1 | 10.2 | 12.1 | 9.2 | 8.5 | 10.0 |
| Total | 1.4 | 1.3 | 1.4 | 3.1 | 3.0 | 3.3 | 2.1 | 2.1 | 2.2 |

Osteoarthritis of hip mild

|  | Men | | | Women | | | Total | | |
| --- | --- | --- | --- | --- | --- | --- | --- | --- | --- |
|  | **%** | **LCI** | **UCI** | **%** | **LCI** | **UCI** | **%** | **LCI** | **UCI** |
| 0-9 | 0.0 | 0.0 | 0.0 | 0.0 | 0.0 | 0.0 | 0.0 | 0.0 | 0.0 |
| 10-19 | 0.0 | 0.0 | 0.0 | 0.1 | 0.1 | 0.1 | 0.1 | 0.0 | 0.1 |
| 20-29 | 0.0 | 0.0 | 0.0 | 0.3 | 0.3 | 0.4 | 0.2 | 0.1 | 0.2 |
| 30-39 | 0.1 | 0.1 | 0.1 | 0.7 | 0.6 | 0.8 | 0.4 | 0.3 | 0.4 |
| 40-49 | 0.2 | 0.1 | 0.2 | 1.4 | 1.2 | 1.6 | 0.8 | 0.7 | 0.9 |
| 50-59 | 0.3 | 0.3 | 0.4 | 2.9 | 2.6 | 3.2 | 1.6 | 1.5 | 1.8 |
| 60-69 | 0.7 | 0.5 | 0.8 | 5.8 | 5.3 | 6.3 | 3.4 | 3.1 | 3.7 |
| 70-79 | 1.4 | 1.1 | 1.7 | 11.4 | 10.4 | 12.4 | 7.0 | 6.4 | 7.6 |
| 80+ | 3.1 | 2.6 | 3.7 | 23.3 | 21.6 | 25.1 | 16.1 | 15.0 | 17.3 |
| Total | 0.3 | 0.2 | 0.3 | 2.9 | 2.7 | 3.0 | 1.5 | 1.4 | 1.5 |

Osteoarthritis of hip moderate

|  | Men | | | Women | | | Total | | |
| --- | --- | --- | --- | --- | --- | --- | --- | --- | --- |
|  | **%** | **LCI** | **UCI** | **%** | **LCI** | **UCI** | **%** | **LCI** | **UCI** |
| 0-9 | 0.0 | 0.0 | 0.0 | 0.0 | 0.0 | 0.0 | 0.0 | 0.0 | 0.0 |
| 10-19 | 0.0 | 0.0 | 0.1 | 0.2 | 0.1 | 0.4 | 0.1 | 0.0 | 0.2 |
| 20-29 | 0.1 | 0.0 | 0.1 | 0.4 | 0.2 | 0.6 | 0.2 | 0.1 | 0.3 |
| 30-39 | 0.1 | 0.0 | 0.1 | 0.3 | 0.2 | 0.5 | 0.2 | 0.1 | 0.3 |
| 40-49 | 0.1 | 0.0 | 0.1 | 0.3 | 0.2 | 0.4 | 0.2 | 0.1 | 0.2 |
| 50-59 | 0.1 | 0.0 | 0.1 | 0.3 | 0.2 | 0.4 | 0.2 | 0.1 | 0.2 |
| 60-69 | 0.0 | 0.0 | 0.1 | 0.3 | 0.2 | 0.4 | 0.2 | 0.1 | 0.2 |
| 70-79 | 0.0 | 0.0 | 0.1 | 0.2 | 0.2 | 0.4 | 0.2 | 0.1 | 0.2 |
| 80+ | 0.0 | 0.0 | 0.1 | 0.2 | 0.1 | 0.4 | 0.2 | 0.1 | 0.2 |
| Total | 0.0 | 0.0 | 0.1 | 0.3 | 0.2 | 0.3 | 0.1 | 0.1 | 0.2 |

*LCI: Lower confidence Interval 95% / UCI: Upper confidence Interval 95%*

Osteoarthritis of hip severe

|  | Men | | | Women | | | Total | | |
| --- | --- | --- | --- | --- | --- | --- | --- | --- | --- |
|  | **%** | **LCI** | **UCI** | **%** | **LCI** | **UCI** | **%** | **LCI** | **UCI** |
| 0-9 | 0.0 | 0.0 | 0.0 | 0.0 | 0.0 | 0.0 | 0.0 | 0.0 | 0.0 |
| 10-19 | 0.0 | 0.0 | 0.0 | 0.1 | 0.0 | 0.1 | 0.0 | 0.0 | 0.0 |
| 20-29 | 0.0 | 0.0 | 0.0 | 0.1 | 0.1 | 0.2 | 0.1 | 0.0 | 0.1 |
| 30-39 | 0.0 | 0.0 | 0.0 | 0.2 | 0.1 | 0.2 | 0.1 | 0.1 | 0.1 |
| 40-49 | 0.0 | 0.0 | 0.0 | 0.3 | 0.2 | 0.3 | 0.1 | 0.1 | 0.2 |
| 50-59 | 0.0 | 0.0 | 0.0 | 0.3 | 0.2 | 0.4 | 0.2 | 0.1 | 0.2 |
| 60-69 | 0.0 | 0.0 | 0.0 | 0.5 | 0.3 | 0.6 | 0.2 | 0.2 | 0.3 |
| 70-79 | 0.0 | 0.0 | 0.0 | 0.6 | 0.4 | 0.8 | 0.3 | 0.2 | 0.5 |
| 80+ | 0.0 | 0.0 | 0.0 | 0.9 | 0.6 | 1.1 | 0.6 | 0.4 | 0.7 |
| Total | 0.0 | 0.0 | 0.0 | 0.3 | 0.2 | 0.3 | 0.1 | 0.1 | 0.1 |

Osteoarthritis of knee mild

|  | Men | | | Women | | | Total | | |
| --- | --- | --- | --- | --- | --- | --- | --- | --- | --- |
|  | **%** | **LCI** | **UCI** | **%** | **LCI** | **UCI** | **%** | **LCI** | **UCI** |
| 0-9 | 0.0 | 0.0 | 0.0 | 0.0 | 0.0 | 0.0 | 0.0 | 0.0 | 0.0 |
| 10-19 | 0.1 | 0.0 | 0.1 | 0.2 | 0.2 | 0.3 | 0.1 | 0.1 | 0.2 |
| 20-29 | 0.2 | 0.2 | 0.3 | 0.7 | 0.6 | 0.9 | 0.5 | 0.4 | 0.5 |
| 30-39 | 0.4 | 0.3 | 0.5 | 1.4 | 1.2 | 1.6 | 0.9 | 0.8 | 1.0 |
| 40-49 | 0.8 | 0.6 | 1.0 | 2.6 | 2.2 | 2.9 | 1.7 | 1.5 | 1.8 |
| 50-59 | 1.4 | 1.1 | 1.8 | 4.7 | 4.2 | 5.2 | 3.1 | 2.8 | 3.4 |
| 60-69 | 2.6 | 2.0 | 3.2 | 8.4 | 7.7 | 9.1 | 5.6 | 5.2 | 6.1 |
| 70-79 | 4.7 | 3.7 | 5.8 | 14.7 | 13.5 | 15.9 | 10.3 | 9.5 | 11.1 |
| 80+ | 9.1 | 7.6 | 10.9 | 26.5 | 24.7 | 28.3 | 20.3 | 19.1 | 21.6 |
| Total | 1.0 | 0.9 | 1.1 | 4.0 | 3.9 | 4.2 | 2.4 | 2.3 | 2.4 |

Osteoarthritis of knee moderate

|  | Men | | | Women | | | Total | | |
| --- | --- | --- | --- | --- | --- | --- | --- | --- | --- |
|  | **%** | **LCI** | **UCI** | **%** | **LCI** | **UCI** | **%** | **LCI** | **UCI** |
| 0-9 | 0.0 | 0.0 | 0.0 | 0.0 | 0.0 | 0.0 | 0.0 | 0.0 | 0.0 |
| 10-19 | 0.1 | 0.0 | 0.1 | 0.1 | 0.1 | 0.2 | 0.1 | 0.1 | 0.1 |
| 20-29 | 0.1 | 0.1 | 0.2 | 0.3 | 0.2 | 0.4 | 0.2 | 0.2 | 0.3 |
| 30-39 | 0.2 | 0.1 | 0.3 | 0.3 | 0.3 | 0.4 | 0.3 | 0.2 | 0.3 |
| 40-49 | 0.2 | 0.1 | 0.3 | 0.4 | 0.3 | 0.5 | 0.3 | 0.3 | 0.4 |
| 50-59 | 0.3 | 0.2 | 0.4 | 0.5 | 0.4 | 0.7 | 0.4 | 0.3 | 0.5 |
| 60-69 | 0.4 | 0.2 | 0.5 | 0.7 | 0.5 | 0.8 | 0.5 | 0.4 | 0.6 |
| 70-79 | 0.4 | 0.3 | 0.6 | 0.9 | 0.7 | 1.1 | 0.7 | 0.5 | 0.8 |
| 80+ | 0.6 | 0.4 | 0.8 | 1.1 | 0.9 | 1.3 | 0.9 | 0.8 | 1.1 |
| Total | 0.2 | 0.2 | 0.2 | 0.4 | 0.4 | 0.5 | 0.3 | 0.3 | 0.3 |

*LCI: Lower confidence Interval 95% / UCI: Upper confidence Interval 95%*

Osteoarthritis of knee severe

|  | Men | | | Women | | | Total | | |
| --- | --- | --- | --- | --- | --- | --- | --- | --- | --- |
|  | **%** | **LCI** | **UCI** | **%** | **LCI** | **UCI** | **%** | **LCI** | **UCI** |
| 0-9 | 0.0 | 0.0 | 0.0 | 0.0 | 0.0 | 0.0 | 0.0 | 0.0 | 0.0 |
| 10-19 | 0.0 | 0.0 | 0.0 | 0.1 | 0.0 | 0.1 | 0.0 | 0.0 | 0.0 |
| 20-29 | 0.0 | 0.0 | 0.0 | 0.1 | 0.1 | 0.2 | 0.1 | 0.1 | 0.1 |
| 30-39 | 0.0 | 0.0 | 0.0 | 0.2 | 0.2 | 0.3 | 0.1 | 0.1 | 0.1 |
| 40-49 | 0.0 | 0.0 | 0.0 | 0.3 | 0.2 | 0.4 | 0.2 | 0.1 | 0.2 |
| 50-59 | 0.0 | 0.0 | 0.0 | 0.5 | 0.4 | 0.6 | 0.2 | 0.2 | 0.3 |
| 60-69 | 0.0 | 0.0 | 0.0 | 0.7 | 0.5 | 0.8 | 0.4 | 0.3 | 0.5 |
| 70-79 | 0.0 | 0.0 | 0.0 | 1.0 | 0.8 | 1.3 | 0.6 | 0.4 | 0.7 |
| 80+ | 0.0 | 0.0 | 0.0 | 1.6 | 1.3 | 1.9 | 1.0 | 0.8 | 1.2 |
| Total | 0.0 | 0.0 | 0.0 | 0.4 | 0.3 | 0.4 | 0.2 | 0.2 | 0.2 |

Fibromyalgia moderate

|  | Men | | | Women | | | Total | | |
| --- | --- | --- | --- | --- | --- | --- | --- | --- | --- |
|  | **%** | **LCI** | **UCI** | **%** | **LCI** | **UCI** | **%** | **LCI** | **UCI** |
| 0-9 | 0.0 | 0.0 | 0.0 | 0.0 | 0.0 | 0.0 | 0.0 | 0.0 | 0.0 |
| 10-19 | 0.1 | 0.1 | 0.2 | 0.3 | 0.1 | 0.6 | 0.2 | 0.1 | 0.3 |
| 20-29 | 0.3 | 0.2 | 0.4 | 0.7 | 0.4 | 1.1 | 0.5 | 0.3 | 0.7 |
| 30-39 | 0.4 | 0.3 | 0.5 | 1.1 | 0.7 | 1.5 | 0.7 | 0.5 | 0.9 |
| 40-49 | 0.6 | 0.4 | 0.8 | 1.6 | 1.2 | 2.0 | 1.1 | 0.9 | 1.3 |
| 50-59 | 0.9 | 0.6 | 1.2 | 2.3 | 1.9 | 2.7 | 1.6 | 1.4 | 1.9 |
| 60-69 | 1.3 | 0.8 | 1.9 | 3.3 | 2.9 | 3.9 | 2.4 | 2.0 | 2.7 |
| 70-79 | 1.9 | 1.0 | 3.0 | 4.9 | 4.0 | 5.9 | 3.6 | 2.9 | 4.3 |
| 80+ | 2.9 | 1.5 | 4.7 | 7.6 | 5.9 | 9.5 | 5.9 | 4.7 | 7.3 |
| Total | 0.5 | 0.5 | 0.6 | 1.8 | 1.6 | 1.9 | 1.1 | 1.0 | 1.2 |

Fibromyalgia severe

|  | Men | | | Women | | | Total | | |
| --- | --- | --- | --- | --- | --- | --- | --- | --- | --- |
|  | **%** | **LCI** | **UCI** | **%** | **LCI** | **UCI** | **%** | **LCI** | **UCI** |
| 0-9 | 0.0 | 0.0 | 0.0 | 0.0 | 0.0 | 0.0 | 0.0 | 0.0 | 0.0 |
| 10-19 | 0.1 | 0.1 | 0.2 | 0.9 | 0.7 | 1.1 | 0.5 | 0.4 | 0.6 |
| 20-29 | 0.3 | 0.3 | 0.4 | 2.1 | 1.8 | 2.5 | 1.2 | 1.1 | 1.4 |
| 30-39 | 0.5 | 0.4 | 0.6 | 2.9 | 2.5 | 3.2 | 1.7 | 1.5 | 1.8 |
| 40-49 | 0.6 | 0.5 | 0.8 | 3.8 | 3.5 | 4.2 | 2.2 | 2.0 | 2.4 |
| 50-59 | 0.8 | 0.7 | 1.0 | 5.1 | 4.7 | 5.5 | 3.0 | 2.8 | 3.2 |
| 60-69 | 1.1 | 0.9 | 1.4 | 6.7 | 6.2 | 7.2 | 4.1 | 3.8 | 4.4 |
| 70-79 | 1.5 | 1.2 | 1.8 | 8.9 | 8.1 | 9.7 | 5.6 | 5.2 | 6.1 |
| 80+ | 2.1 | 1.7 | 2.5 | 12.1 | 11.0 | 13.3 | 8.6 | 7.8 | 9.3 |
| Total | 0.5 | 0.5 | 0.6 | 3.8 | 3.7 | 4.0 | 2.0 | 2.0 | 2.1 |

*LCI: Lower confidence Interval 95% / UCI: Upper confidence Interval 95%*

Chronic musculoskeletal pain mild

|  | Men | | | Women | | | Total | | |
| --- | --- | --- | --- | --- | --- | --- | --- | --- | --- |
|  | **%** | **LCI** | **UCI** | **%** | **LCI** | **UCI** | **%** | **LCI** | **UCI** |
| 0-9 | 0.0 | 0.0 | 0.0 | 0.0 | 0.0 | 0.0 | 0.0 | 0.0 | 0.0 |
| 10-19 | 0.8 | 0.5 | 1.3 | 0.4 | 0.2 | 0.5 | 0.6 | 0.4 | 0.9 |
| 20-29 | 1.7 | 1.2 | 2.2 | 0.7 | 0.6 | 0.9 | 1.2 | 1.0 | 1.5 |
| 30-39 | 1.7 | 1.3 | 2.2 | 0.8 | 0.6 | 0.9 | 1.3 | 1.0 | 1.5 |
| 40-49 | 1.8 | 1.5 | 2.1 | 0.8 | 0.6 | 1.0 | 1.3 | 1.1 | 1.5 |
| 50-59 | 1.8 | 1.5 | 2.2 | 0.8 | 0.6 | 1.0 | 1.3 | 1.1 | 1.5 |
| 60-69 | 1.9 | 1.5 | 2.3 | 0.8 | 0.6 | 1.1 | 1.3 | 1.1 | 1.6 |
| 70-79 | 2.0 | 1.5 | 2.5 | 0.9 | 0.6 | 1.2 | 1.4 | 1.1 | 1.7 |
| 80+ | 2.0 | 1.5 | 2.7 | 0.9 | 0.6 | 1.3 | 1.3 | 1.0 | 1.7 |
| Total | 1.4 | 1.3 | 1.6 | 0.7 | 0.6 | 0.8 | 1.0 | 0.9 | 1.1 |

Chronic musculoskeletal pain moderate

|  | Men | | | Women | | | Total | | |
| --- | --- | --- | --- | --- | --- | --- | --- | --- | --- |
|  | **%** | **LCI** | **UCI** | **%** | **LCI** | **UCI** | **%** | **LCI** | **UCI** |
| 0-9 | 0.0 | 0.0 | 0.0 | 0.0 | 0.0 | 0.0 | 0.0 | 0.0 | 0.0 |
| 10-19 | 3.1 | 2.6 | 3.6 | 3.9 | 3.3 | 4.7 | 3.5 | 3.1 | 4.0 |
| 20-29 | 6.6 | 6.0 | 7.3 | 8.4 | 7.6 | 9.3 | 7.5 | 7.0 | 8.1 |
| 30-39 | 7.6 | 6.9 | 8.3 | 9.6 | 8.8 | 10.4 | 8.6 | 8.1 | 9.1 |
| 40-49 | 8.7 | 8.0 | 9.5 | 11.0 | 10.3 | 11.7 | 9.9 | 9.4 | 10.4 |
| 50-59 | 10.0 | 9.1 | 10.9 | 12.5 | 11.8 | 13.2 | 11.3 | 10.7 | 11.9 |
| 60-69 | 11.4 | 10.2 | 12.6 | 14.2 | 13.4 | 15.1 | 12.9 | 12.2 | 13.6 |
| 70-79 | 12.9 | 11.4 | 14.5 | 16.1 | 15.0 | 17.3 | 14.7 | 13.8 | 15.7 |
| 80+ | 14.9 | 13.1 | 16.8 | 18.6 | 17.2 | 20.1 | 17.3 | 16.2 | 18.5 |
| Total | 6.9 | 6.6 | 7.1 | 9.8 | 9.6 | 10.1 | 8.0 | 7.8 | 8.2 |

Chronic musculoskeletal pain severe

|  | Men | | | Women | | | Total | | |
| --- | --- | --- | --- | --- | --- | --- | --- | --- | --- |
|  | **%** | **LCI** | **UCI** | **%** | **LCI** | **UCI** | **%** | **LCI** | **UCI** |
| 0-9 | 0.0 | 0.0 | 0.0 | 0.0 | 0.0 | 0.0 | 0.0 | 0.0 | 0.0 |
| 10-19 | 1.6 | 1.4 | 1.8 | 4.4 | 3.9 | 4.9 | 3.0 | 2.7 | 3.2 |
| 20-29 | 3.7 | 3.4 | 4.1 | 10.1 | 9.4 | 10.8 | 6.9 | 6.5 | 7.3 |
| 30-39 | 4.8 | 4.4 | 5.2 | 12.7 | 12.0 | 13.4 | 8.7 | 8.3 | 9.1 |
| 40-49 | 6.1 | 5.6 | 6.6 | 15.9 | 15.2 | 16.6 | 11.0 | 10.6 | 11.5 |
| 50-59 | 7.8 | 7.1 | 8.4 | 19.7 | 18.9 | 20.5 | 13.9 | 13.4 | 14.4 |
| 60-69 | 9.8 | 9.0 | 10.6 | 24.0 | 23.0 | 25.1 | 17.3 | 16.7 | 18.0 |
| 70-79 | 12.3 | 11.2 | 13.4 | 29.1 | 27.8 | 30.5 | 21.8 | 20.9 | 22.7 |
| 80+ | 15.8 | 14.5 | 17.1 | 35.7 | 34.2 | 37.2 | 28.6 | 27.5 | 29.7 |
| Total | 5.0 | 4.8 | 5.1 | 14.7 | 14.4 | 15.0 | 9.3 | 9.2 | 9.5 |

*LCI: Lower confidence Interval 95% / UCI: Upper confidence Interval 95%*

**Table S6**. Disability Adjusted Life Years for selected musculoskeletal disorders for Chile, 2017

Chronic low back pain

|  | Men | | | Women | | | Total | | |
| --- | --- | --- | --- | --- | --- | --- | --- | --- | --- |
|  | **DALY** | **LCI** | **UCI** | **DALY** | **LCI** | **UCI** | **DALY** | **LCI** | **UCI** |
| 0-9 | 0 | 0 | 0 | 0 | 0 | 0 | 0 | 0 | 0 |
| 10-19 | 4.299 | 1.312 | 9.549 | 7.174 | 1.987 | 15.862 | 11.473 | 4.903 | 21.128 |
| 20-29 | 10.027 | 2.655 | 22.939 | 17.032 | 3.901 | 40.128 | 27.059 | 10.381 | 52.263 |
| 30-39 | 9.229 | 2.308 | 21.599 | 16.349 | 3.456 | 40.231 | 25.578 | 9.545 | 51.528 |
| 40-49 | 8.936 | 2.147 | 21.570 | 16.010 | 3.211 | 40.017 | 24.947 | 8.870 | 51.148 |
| 50-59 | 8.302 | 1.969 | 20.740 | 14.791 | 2.933 | 38.255 | 23.093 | 8.173 | 48.241 |
| 60-69 | 5.959 | 1.346 | 14.603 | 11.090 | 2.114 | 28.621 | 17.049 | 5.817 | 35.966 |
| 70-79 | 3.218 | 742 | 8.254 | 5.948 | 1.139 | 15.510 | 9.166 | 3.143 | 19.403 |
| 80+ | 1.564 | 335 | 4.016 | 2.870 | 521 | 7.546 | 4.434 | 1.472 | 9.549 |
| Total | 51.533 | 32.451 | 76.834 | 91.264 | 55.432 | 137.086 | 142.798 | 100.728 | 194.089 |

Chronic shoulder pain

|  | Men | | | Women | | | Total | | |
| --- | --- | --- | --- | --- | --- | --- | --- | --- | --- |
|  | **DALY** | **LCI** | **UCI** | **DALY** | **LCI** | **UCI** | **DALY** | **LCI** | **UCI** |
| 0-9 | 0 | 0 | 0 | 0 | 0 | 0 | 0 | 0 | 0 |
| 10-19 | 485 | 45 | 1.494 | 945 | 66 | 3.066 | 1.430 | 277 | 3.650 |
| 20-29 | 1.430 | 84 | 4.774 | 2.768 | 117 | 9.429 | 4.198 | 651 | 11.329 |
| 30-39 | 1.804 | 87 | 6.209 | 3.414 | 127 | 12.231 | 5.218 | 722 | 14.803 |
| 40-49 | 2.281 | 108 | 8.113 | 4.291 | 132 | 15.582 | 6.572 | 868 | 18.244 |
| 50-59 | 2.763 | 120 | 10.050 | 5.399 | 156 | 20.347 | 8.162 | 994 | 23.934 |
| 60-69 | 2.632 | 103 | 9.495 | 4.945 | 140 | 18.344 | 7.577 | 896 | 21.908 |
| 70-79 | 1.816 | 64 | 6.534 | 3.344 | 87 | 12.873 | 5.160 | 580 | 15.566 |
| 80+ | 1.154 | 39 | 4.206 | 2.099 | 44 | 8.011 | 3.253 | 349 | 9.585 |
| Total | 14.365 | 6.011 | 26.668 | 27.205 | 11.215 | 51.106 | 41.570 | 22.587 | 67.371 |

*DALY: disability adjusted life years / LCI: Lower confidence Interval 95% / UCI: Upper confidence Interval 95%*

Osteoarthritis of Hip

|  | Men | | | Women | | | Total | | |
| --- | --- | --- | --- | --- | --- | --- | --- | --- | --- |
|  | **DALY** | **LCI** | **UCI** | **DALY** | **LCI** | **UCI** | **DALY** | **LCI** | **UCI** |
| 0-9 | 0 | 0 | 0 | 0 | 0 | 0 | 0 | 0 | 0 |
| 10-19 | 39 | 10 | 91 | 344 | 122 | 685 | 383 | 156 | 727 |
| 20-29 | 85 | 21 | 203 | 879 | 268 | 1.853 | 964 | 341 | 1.942 |
| 30-39 | 85 | 26 | 184 | 974 | 296 | 2.063 | 1.059 | 372 | 2.165 |
| 40-49 | 96 | 29 | 204 | 1.185 | 329 | 2.623 | 1.281 | 416 | 2.715 |
| 50-59 | 127 | 32 | 300 | 1.556 | 371 | 3.702 | 1.683 | 478 | 3.868 |
| 60-69 | 144 | 25 | 406 | 1.666 | 332 | 4.551 | 1.810 | 449 | 4.682 |
| 70-79 | 131 | 16 | 421 | 1.398 | 206 | 4.254 | 1.529 | 310 | 4.376 |
| 80+ | 129 | 9 | 453 | 1.163 | 136 | 3.787 | 1.292 | 220 | 3.915 |
| Total | 835 | 488 | 1.338 | 9.166 | 5.572 | 14.117 | 10.001 | 6.361 | 14.942 |

Osteoarthritis of Knee

|  | Men | | | Women | | | Total | | |
| --- | --- | --- | --- | --- | --- | --- | --- | --- | --- |
|  | **DALY** | **LCI** | **UCI** | **DALY** | **LCI** | **UCI** | **DALY** | **LCI** | **UCI** |
| 0-9 | 0 | 0 | 0 | 0 | 0 | 0 | 0 | 0 | 0 |
| 10-19 | 86 | 26 | 190 | 306 | 116 | 593 | 391 | 184 | 689 |
| 20-29 | 251 | 61 | 599 | 934 | 282 | 1.960 | 1.185 | 469 | 2.246 |
| 30-39 | 320 | 78 | 724 | 1.275 | 357 | 2.767 | 1.595 | 610 | 3.128 |
| 40-49 | 442 | 96 | 1.053 | 1.767 | 442 | 4.146 | 2.209 | 783 | 4.618 |
| 50-59 | 594 | 115 | 1.525 | 2.419 | 564 | 6.015 | 3.013 | 986 | 6.635 |
| 60-69 | 659 | 108 | 1.801 | 2.651 | 509 | 7.034 | 3.310 | 1.005 | 7.788 |
| 70-79 | 538 | 78 | 1.595 | 2.087 | 379 | 5.890 | 2.625 | 724 | 6.501 |
| 80+ | 444 | 51 | 1.423 | 1.542 | 245 | 4.550 | 1.986 | 484 | 5.040 |
| Total | 3.333 | 1.949 | 5.234 | 12.982 | 7.806 | 20.183 | 16.315 | 10.924 | 23.634 |

Fibromyalgia

|  | Men | | | Women | | | Total | | |
| --- | --- | --- | --- | --- | --- | --- | --- | --- | --- |
|  | **DALY** | **LCI** | **UCI** | **DALY** | **LCI** | **UCI** | **DALY** | **LCI** | **UCI** |
| 0-9 | 0 | 0 | 0 | 0 | 0 | 0 | 0 | 0 | 0 |
| 10-19 | 1.433 | 350 | 3.379 | 7.609 | 1.193 | 22.402 | 9.042 | 2.326 | 23.842 |
| 20-29 | 4.269 | 833 | 11.006 | 21.221 | 2.549 | 67.046 | 25.490 | 5.841 | 70.526 |
| 30-39 | 5.319 | 932 | 13.591 | 26.046 | 2.719 | 84.603 | 31.365 | 6.540 | 89.702 |
| 40-49 | 6.576 | 1.155 | 17.442 | 31.874 | 3.127 | 104.448 | 38.450 | 8.040 | 111.713 |
| 50-59 | 8.108 | 1.299 | 21.141 | 37.701 | 3.506 | 125.741 | 45.809 | 9.161 | 134.968 |
| 60-69 | 7.675 | 1.302 | 20.259 | 35.122 | 3.100 | 113.279 | 42.797 | 8.430 | 121.563 |
| 70-79 | 5.340 | 897 | 14.153 | 23.251 | 2.282 | 75.427 | 28.591 | 6.068 | 81.687 |
| 80+ | 3.509 | 600 | 9.200 | 14.213 | 1.479 | 45.007 | 17.721 | 3.973 | 48.513 |
| Total | 42.228 | 24.550 | 65.184 | 197.037 | 97.828 | 344.555 | 239.265 | 138.883 | 388.334 |

*DALY: disability adjusted life years / LCI: Lower confidence Interval 95% / UCI: Upper confidence Interval 95%*

Chronic musculoskeletal pain

|  | Men | | | Women | | | Total | | |
| --- | --- | --- | --- | --- | --- | --- | --- | --- | --- |
|  | **DALY** | **LCI** | **UCI** | **DALY** | **LCI** | **UCI** | **DALY** | **LCI** | **UCI** |
| 0-9 | 0 | 0 | 0 | 0 | 0 | 0 | 0 | 0 | 0 |
| 10-19 | 7.908 | 1.158 | 23.165 | 17.778 | 1.556 | 59.904 | 25.686 | 5.742 | 69.143 |
| 20-29 | 21.409 | 2.480 | 65.795 | 48.208 | 3.905 | 168.111 | 69.617 | 13.737 | 195.451 |
| 30-39 | 23.722 | 2.621 | 72.827 | 53.718 | 3.919 | 190.671 | 77.440 | 14.888 | 217.282 |
| 40-49 | 26.623 | 2.600 | 85.232 | 59.567 | 4.099 | 220.779 | 86.190 | 15.955 | 253.776 |
| 50-59 | 28.763 | 2.723 | 94.338 | 65.679 | 3.999 | 239.994 | 94.442 | 16.335 | 271.310 |
| 60-69 | 24.454 | 2.058 | 79.389 | 53.359 | 3.083 | 193.139 | 77.814 | 13.119 | 224.205 |
| 70-79 | 15.236 | 1.336 | 51.335 | 31.718 | 1.730 | 117.200 | 46.954 | 8.100 | 136.395 |
| 80+ | 8.561 | 697 | 29.079 | 17.216 | 953 | 65.859 | 25.777 | 4.414 | 75.718 |
| Total | 156.675 | 78.422 | 271.049 | 347.244 | 155.117 | 640.074 | 503.919 | 283.940 | 815.132 |

*DALY: disability adjusted life years / LCI: Lower confidence Interval 95% / UCI: Upper confidence Interval 95%*

**Table S7**. Fraction of loss of health state utilities attributable to domains of the EQ5D questionnaire for general population and selected musculoskeletal disorders, using data from the Chilean National Health Survey 2016-2017 (n=5,077)

|  | **General population** | | |  | **Chronic low back pain** | | |
| --- | --- | --- | --- | --- | --- | --- | --- |
|  | % | LCI | UCI |  | % | LCI | UCI |
| Mobility | 8.2 | 7.2 | 9.3 |  | 5.4 | 3.3 | 7.4 |
| Self care | 1.0 | 0.8 | 1.2 |  | 1.0 | 0.3 | 1.6 |
| Usual activities | 4.4 | 3.8 | 5.0 |  | 7.8 | 4.3 | 11.4 |
| Pain & Discomfort | 53.6 | 51.2 | 56.1 |  | 57.8 | 48.2 | 67.4 |
| Anxiety/ Depression | 21.5 | 19.4 | 23.5 |  | 17.2 | 9.7 | 24.8 |
|  |  |  |  |  |  |  |  |
|  |  |  |  |  |  |  |  |
|  | **Chronic shoulder pain** | | |  | **Osteoarthritis of hip** | | |
|  | % | LCI | UCI |  | % | LCI | UCI |
| Mobility | 7.6 | 4.9 | 10.2 |  | 13.9 | 10.5 | 17.4 |
| Self care | 1.0 | 0.3 | 1.7 |  | 2.6 | 1.4 | 3.9 |
| Usual activities | 5.3 | 2.9 | 7.7 |  | 9.3 | 6.4 | 12.3 |
| Pain & Discomfort | 54.5 | 44.2 | 64.7 |  | 49.0 | 40.7 | 57.3 |
| Anxiety/ Depression | 19.7 | 10.8 | 28.7 |  | 12.3 | 8.7 | 15.9 |
|  |  |  |  |  |  |  |  |
|  | **Osteoarthritis of knee** | | |  | **Fibromyalgia** | | |
|  | % | LCI | UCI |  | % | LCI | UCI |
| Mobility | 14.6 | 11.9 | 17.3 |  | 11.7 | 9.2 | 14.1 |
| Self care | 2.4 | 1.1 | 3.7 |  | 2.5 | 1.4 | 3.5 |
| Usual activities | 9.7 | 6.8 | 12.6 |  | 8.7 | 6.4 | 10.9 |
| Pain & Discomfort | 43.8 | 36.2 | 51.4 |  | 42.2 | 35.9 | 48.4 |
| Anxiety/ Depression | 16.3 | 11.2 | 21.5 |  | 20.4 | 17.0 | 23.9 |
|  |  |  |  |  |  |  |  |
|  | **Chronic Musculoskeletal pain** | | |  |  |  |  |
|  | % | LCI | UCI |  |  |  |  |
| Mobility | 8.3 | 7.2 | 9.4 |  |  |  |  |
| Self care | 1.4 | 1.0 | 1.9 |  |  |  |  |
| Usual activities | 6.3 | 5.0 | 7.7 |  |  |  |  |
| Pain & Discomfort | 52.8 | 48.9 | 56.8 |  |  |  |  |
| Anxiety/ Depression | 18.0 | 14.8 | 21.2 |  |  |  |  |

*LCI: Lower confidence Interval 95% / UCI: Upper confidence Interval 95%*

**Table S8**. Health state utilities attributable, in people with and without selected musculoskeletal conditions, using data from the Chilean National Health Survey 2016-2017 (n=5,077)

|  | HSU atributable | | | HSU without disease | | | HSU with disease | | |
| --- | --- | --- | --- | --- | --- | --- | --- | --- | --- |
|  | HSU | LCI | UCI | HSU | LCI | UCI | HSU | LCI | UCI |
| Chronic low back pain | 0.055 | 0.018 | 0.091 | 0.782 | 0.791 | 0.772 | 0.696 | 0.730 | 0.661 |
| Chronic shoulder pain | 0.004 | -0.065 | 0.074 | 0.778 | 0.788 | 0.769 | 0.681 | 0.749 | 0.614 |
| Osteoarthritis of Hip | 0.074 | -0.001 | 0.149 | 0.781 | 0.791 | 0.772 | 0.503 | 0.565 | 0.440 |
| Osteoarthritis of Knee | 0.101 | 0.031 | 0.173 | 0.785 | 0.794 | 0.776 | 0.515 | 0.579 | 0.450 |
| Fibromyalgia | 0.232 | 0.169 | 0.295 | 0.790 | 0.800 | 0.781 | 0.418 | 0.477 | 0.358 |
| Chronic Musculoskeletal pain | 0.099 | 0.072 | 0.127 | 0.811 | 0.821 | 0.800 | 0.650 | 0.674 | 0.627 |

*LCI: Lower confidence Interval 95% / UCI: Upper confidence Interval 95%*

*HSU: Health State Utilities*

*HSU are anchored in values 0 and 1, equivalent to death and perfect health, respectively.*
